# Supplementary material for: Russula orientalovirescens sp. nov., a common Southeast Asian edible fungus is different from the European look-alike R. virescens
Source: PLoS One. 2025 Aug 20;20(8):e0322545. doi: 10.1371/journal.pone.0322545 (PMC12367157; doi:10.1371/journal.pone.0322545)
Supplement: Attachment — Submitted filename: Summary of Comments on PONE-D-24-43558_reviewer.pdf [file pone.0322545.s002.pdf]

PLOS ONE

Russula orientalovirescens sp. nov., a common Southeast Asian edible fungus is different from the European look-alike R. virescens

--Manuscript Draft--

**Manuscript Number:**	PONE-D-24-43558
**Article Type:**	Research Article
**Full Title:**	Russula orientalovirescens sp. nov., a common Southeast Asian edible fungus is different from the European look-alike R. virescens
**Short Title:**	Russula orientalovirescens sp. nov., a new edible taxon from Southeast Asia
**Corresponding Author:**	Olivier Raspé<br>Mae Fah Luang University School of Science<br>Chiang Rai, THAILAND
**Keywords:**	Russulaceae; diversity; Taxonomy; edible mushroom; ethnomycology
**Abstract:**	Green-cracking Russulas are edible fungi that are widely consumed and traded in Southeast Asia. Asian collections of this morphotype were frequently identified as R. virescens in local literature. Multilocus phylogenetic analyses of ITS nrDNA, rpb2 and tef1 regions presented in this study strongly supported that the majority of green cracking Russula collections from South east Asia represent species different from European R. virescens and these collections are described here as R.orientalovirescens sp. nova. Analysis of ITS barcoding region confirmed that published sequence data from China, Laos and Myanmar reported this species as R. virescens. In addition, this analysis showed that the species is widely distributed in Southeast Asia from Malayan Peninsula to Japan, is preferring areas with dry season and is associated with coniferous and deciduous trees as well as heterotrophic plants. Morphological analyses and detailed comparison with recent collections of R. virescens showed that R. orientalovirescens differs from the latter by larger spores and shorter and more abundant pileocystidia. Green cracking Russula species with distinctly areolate pileus formed a monophyletic lineage where our new species is grouped with Asian R. viridirubrolimbata, European R. virescens and North American R. parvovirescens. Few publicly available ITS sequences from Southeast Asia clustered with either European or North American species suggesting that the phylogenetic lineage of green-cracking Russulas urgently require further attention.
**Order of Authors:**	Komsit Wisitrassameewong, Ph.D.<br>Slavomír Adamčík, PhD.<br>Katarína Adamčíková<br>Song-Ming Tang, PhD.<br>Boontiya Chuankid, PhD.<br>Olivier Raspé, PhD.
**Additional Information:**	
**Question**	**Response**
**Financial Disclosure**<br><br>Enter a financial disclosure statement that describes the sources of funding for the work included in this submission. Review the submission guidelines for detailed requirements. View published research articles from *PLOS ONE* for specific examples.	Yes

Summary of comments: PONE-D-24-43558_reviewer.pdf

No Comments.

This statement is required for submission and **will appear in the published article** if the submission is accepted. Please make sure it is accurate.<br><br>**Funded studies**<br>Enter a statement with the following details:<br>• Initials of the authors who received each award<br>• Grant numbers awarded to each author<br>• The full name of each funder<br>• URL of each funder website<br>• Did the sponsors or funders play any role in the study design, data collection and analysis, decision to publish, or preparation of the manuscript?<br><br>Did you receive funding for this work?	
Please add funding details.<br>as follow-up to "**Financial Disclosure**<br><br>Enter a financial disclosure statement that describes the sources of funding for the work included in this submission. Review the submission guidelines for detailed requirements. View published research articles from *PLOS ONE* for specific examples.<br><br>This statement is required for submission and **will appear in the published article** if the submission is accepted. Please make sure it is accurate.<br><br>**Funded studies**<br>Enter a statement with the following details:<br>• Initials of the authors who received each award<br>• Grant numbers awarded to each author<br>• The full name of each funder<br>• URL of each funder website<br>• Did the sponsors or funders play any role in the study design, data collection and analysis, decision to publish, or preparation of the manuscript?	O. Raspé: Mae Fah Luang University grant 641A01003, "Survey of edible fungi in dry dipterocarp forests of Chiang Mai Province, Thailand<br><br>Komsit Wisitrassameewong: the Ecological Monitoring and Plant Specimen and Barcode References project P2250745, National Science and Technology Development Agency<br><br>Slavomír Adamčík, Katarína Adamčíková: the Slovak Research and Development Agency projects APVV-15-0210 and APVV-20-0257.

No Comments.

Did you receive funding for this work?"	
Please select the country of your main research funder (please select carefully as in some cases this is used in fee calculation).<br>as follow-up to "**Financial Disclosure**<br><br>Enter a financial disclosure statement that describes the sources of funding for the work included in this submission. Review the submission guidelines for detailed requirements. View published research articles from *PLOS ONE* for specific examples.<br><br>This statement is required for submission and **will appear in the published article** if the submission is accepted. Please make sure it is accurate.<br><br>**Funded studies**<br>Enter a statement with the following details:<br>• Initials of the authors who received each award<br>• Grant numbers awarded to each author<br>• The full name of each funder<br>• URL of each funder website<br>• Did the sponsors or funders play any role in the study design, data collection and analysis, decision to publish, or preparation of the manuscript?<br><br>Did you receive funding for this work?"	THAILAND - TH
**Competing Interests**<br><br>Use the instructions below to enter a competing interest statement for this submission. On behalf of all authors, disclose any competing interests that could be perceived to bias this work—acknowledging all financial support and any other relevant financial or non-financial competing interests.<br><br>This statement is required for submission and **will appear in the published article** if	The authors declare no competing interests.

No Comments.

the submission is accepted. Please make sure it is accurate and that any funding sources listed in your Funding Information later in the submission form are also declared in your Financial Disclosure statement.<br><br>View published research articles from *PLOS ONE* for specific examples.<br><br>**NO authors have competing interests**<br><br>Enter: *The authors have declared that no competing interests exist.*<br><br>**Authors with competing interests**<br><br>Enter competing interest details beginning with this statement:<br><br>*I have read the journal's policy and the authors of this manuscript have the following competing interests: [insert competing interests here]*<br><br>* typeset	
**Ethics Statement**<br><br>Enter an ethics statement for this submission. This statement is required if the study involved:<br><br>• Human participants<br>• Human specimens or tissue<br>• Vertebrate animals or cephalopods<br>• Vertebrate embryos or tissues<br>• Field research<br><br>Write "N/A" if the submission does not require an ethics statement.<br><br>General guidance is provided below. Consult the submission guidelines for detailed instructions. **Make sure that all information entered here is included in the Methods section of the manuscript.**	"N/A"<br><br>O. Raspé: Mae Fah Luang University grant 641A01003, “Survey of edible fungi in dry dipterocarp forests of Chiang Mai Province, Thailand<br><br>Slavomír Adamčík, Katarína Adamčíková: the Slovak Research and Development Agency projects APVV-15-0210 and APVV-20-0257.

No Comments.

**Format for specific study types**<br><br>**Human Subject Research (involving human participants and/or tissue)**<br>• Give the name of the institutional review board or ethics committee that approved the study<br>• Include the approval number and/or a statement indicating approval of this research<br>• Indicate the form of consent obtained (written/oral) or the reason that consent was not obtained (e.g. the data were analyzed anonymously)<br><br>**Animal Research (involving vertebrate animals, embryos or tissues)**<br>• Provide the name of the Institutional Animal Care and Use Committee (IACUC) or other relevant ethics board that reviewed the study protocol, and indicate whether they approved this research or granted a formal waiver of ethical approval<br>• Include an approval number if one was obtained<br>• If the study involved *non-human primates*, add *additional details* about animal welfare and steps taken to ameliorate suffering<br>• If anesthesia, euthanasia, or any kind of animal sacrifice is part of the study, include briefly which substances and/or methods were applied<br><br>**Field Research**<br><br>Include the following details if this study involves the collection of plant, animal, or other materials from a natural setting:<br>• Field permit number<br>• Name of the institution or relevant body that granted permission	
**Data Availability**<br><br>Authors are required to make all data underlying the findings described fully available, without restriction, and from the time of publication. PLOS allows rare exceptions to address legal and ethical concerns. See the PLOS Data Policy and FAQ for detailed information.	Yes - all data are fully available without restriction

No Comments.

A Data Availability Statement describing where the data can be found is required at submission. Your answers to this question constitute the Data Availability Statement and **will be published in the article**, if accepted.<br><br>**Important:** Stating 'data available on request from the author' is not sufficient. If your data are only available upon request, select 'No' for the first question and explain your exceptional situation in the text box.<br><br>Do the authors confirm that all data underlying the findings described in their manuscript are fully available without restriction?	
**Describe where the data may be found in full sentences. If you are copying our sample text, replace any instances of XXX with the appropriate details.**<br><br>• If the data are **held or will be held in a public repository**, include URLs, accession numbers or DOIs. If this information will only be available after acceptance, indicate this by ticking the box below. For example: *All XXX files are available from the XXX database (accession number(s) XXX, XXX.*).<br>• If the data are all contained **within the manuscript and/or Supporting Information files**, enter the following: *All relevant data are within the manuscript and its Supporting Information files.*<br>• If neither of these applies but you are able to provide **details of access elsewhere**, with or without limitations, please do so. For example:<br><br>*Data cannot be shared publicly because of [XXX]. Data are available from the XXX Institutional Data Access / Ethics Committee (contact via XXX) for researchers who meet the criteria for access to confidential data.*<br><br>*The data underlying the results presented in the study are available from (include the name of the third party*	All relevant data are within the manuscript and its Supporting Information files.

Powered by Editorial Manager® and ProduXion Manager® from Aries Systems Corporation

No Comments.

and contact information or URL).<br>• This text is appropriate if the data are owned by a third party and authors do not have permission to share the data.<br><br>* typeset	
Additional data availability information:	Tick here if the URLs/accession numbers/DOIs will be available only after acceptance of the manuscript for publication so that we can ensure their inclusion before publication.

Powered by Editorial Manager® and ProduXion Manager® from Aries Systems Corporation

No Comments.

Manuscript

Click here to access/download;Manuscript;Manuscript - Russula orientalovirescens sp nov.docx

Russula orientalovirescens sp. nov., a common Southeast Asian edible fungus is different from European look-alike *R. virescens*

Komsit Wisitrassameewong[1,2], Slavomír Adamčík[3,4], Katarína Adamčíková[3,5], Song-Ming Tang[6], Boontiya Chuankid[7], Olivier Raspé[7,*]

1 Department of Biotechnology, Faculty of Science, Mahidol University, Bangkok 10400, Thailand; komsit.wis@mahidol.ac.th, https://orcid.org/0000-0003-1195-0338

2 National Biobank of Thailand, National Science and Technology Development (NSTDA), Thailand Science Park, Khlong Neung, Khlong Luang, Pathum Thani 12120, Thailand

3 Laboratory of Molecular Ecology and Mycology, Institute of Botany, Plant Science and Biodiversity Center, Slovak Academy of Sciences, Dúbravská cesta 9, Bratislava 84523, Slovakia; slavomir.adamcik@savba.sk, https://orcid.org/0000-0003-2156-5767

4 Department of Botany, Faculty of Natural Sciences, Comenius University in Bratislava, Révová 39, Bratislava 81102, Slovakia

5 Department of Plant Pathology and Mycology, Institute of Forest Ecology, Slovak Academy of Sciences Zvolen, Akademická 2, Nitra 94901, Slovakia; katarina.adamcikova@ife.sk, https://orcid.org/0000-0001-5407-5233

6 College of Agriculture and Biological Science, Dali University, Dali 671003, China

7 School of Science, Mae Fah Luang University, Chiang Rai, Thailand

*Author for correspondence: olivier.ras@mfu.ac.th

Abstract

Green-cracking Russulas are edible fungi that are widely consumed and traded in Southeast Asia. Asian collections of this morphotype were frequently identified as *R. virescens* in local literature. Multilocus phylogenetic analyses of ITS nrDNA, *rpb*2 and *tef*1 regions presented in this study strongly supported that the majority of green cracking *Russula* collections from South east Asia represent species different from European *R. virescens* and these collections are described here as *R. orientalovirescens* sp. nova. Analysis of ITS barcoding region confirmed that published sequence data from China, Laos and Myanmar reported this species as *R. virescens.* In addition, this analysis showed that the species is widely distributed in Southeast Asia from Malayan Peninsula to Japan, is preferring areas with dry season and is associated with coniferous and deciduous trees as well as heterotrophic plants. Morphological analyses and detailed comparison with recent collections of *R. virescens* showed that *R. orientalovirescens* differs from the latter by larger spores and shorter and more abundant pileocystidia. Green cracking *Russula* species with distinctly areolate pileus formed a monophyletic lineage where our new species is grouped with Asian *R. viridirubrolimbata*, European *R. virescens* and North American *R. parvovirescens*. Few publicly available ITS sequences from Southeast Asia clustered with either European or North American species suggesting that the phylogenetic lineage of green-cracking Russulas urgently require further attention.

Keywords: Russulaceae, diversity, taxonomy, edible mushroom, ethnomycology

No Comments.

Introduction

Russula is a genus of agarics (fungi, Agaricomycotina, Russulales) of high ecological importance because its members are very diverse ectomycorrhizal root symbionts of trees and dominate several types of ectotrophic forest soil ecosystems (Corrales et al. 2018). Among hundreds of described species, there are only few species collected for food and traded and this economic activity is especially popular in Southeast Asia (Sanmee et al. 2003, Wang 2020, Anh et al. 2023). Among the most common edible and traded fungi of Thailand are green-cracking Russulas which are recognized and named as *R. virescens* (Schaeff.) Fr. in local scientific and professional literature (Høiland and Schumacher 1982, Manassila et al. 2005, Chandrasrikul et al. 2008). *Russula virescens* has been known as an edible fungus for centuries all over Europe and has various popular names in different languages and dialects (Gillet 1876, Singer 1932). Its popularity is bolstered by easy identification, it is the only green areolate *Russula* in the European subcontinent (Romagnesi 1967, Sarnari 1998). The species originally described from Europe was reported also from other continents, specifically from the United States (Morgan 1883, Peck 1872, Burlingham 1915, Murrill 1916, Beardslee 1918, Shaffer 1970), Central America (Gómez and Alfaro 1996) and Southeast Asia (Chandrasrikul et al. 2008).

Russula virescens is the type species of *Russula* subsection *Virescentinae* Singer, the group defined by usually areolate pileus surface and typical "virescens-type" structure of pileipellis formed by longer subulate terminal cells originating from one or multiple shorter ellipsoid or globular elements forming almost an epithelium (Romagnesi 1967). However, recent phylogenies suggest that areolate pileus is not a diagnostic character and the phylogenetic lineage of *R. virescens* includes also *R. mustelina* Fr., a species without areolate pileus (Buyck et al. 2018). This group is classified in *R.* subgenus *Heterophyllidia* Romagn. and bears some typical characters of this subgenus like the absence of suprahilar amyloid spot on spores, and one-celled pileocystidia with weak reaction to sulfovanillin and a single apical knob (Buyck and Adamčík 2011).

Soon it became apparent that at least part of so-called *R. virescens* in areas distant from Europe represent different species, and *R. parvovirescens* Buyck, D. Mitch. & Parrent was described as the first look-alike species from the United States (Buyck et al. 2006). Meanwhile, among seventeen *Virescentinae* species reported from China, India, Japan, Pakistan, Papua New Guinea and Thailand (Chen et al. 2019 and 2021, Das et al. 2017, Deng et al. 2020, Han et al. 2023, Hongo 1979, Hyde et al. 2016 and 2019, Li 2014, Paloi et al. 2023, Song et al. 2018, Ullah et al. 2020), only four have similar colour to *R. virescens*. These green Southeast Asian species, *R. prasina* G.J. Li & R.L. Zhao (Hyde et al. 2019), *R. xanthovirens* Y. Song & L.H. Qiu (Song et al. 2018), *R. aureoviridis* Jing W. Li & L.H. Qiu (Das et al. 2017) and *R. sribuabanensis* Paloi, Suwannarach N. & Kumla J. (Paloi et al. 2023) have cuticle which is not distinctly areolate and is only cracking near the pileus margin. All collections of green distinctly areolate Russulas from Southeast Asia were so far identified as *R. virescens* or as *R.* cf. *virescens*, even when using in deep and specifically focused phylogenetic analyses (Cao et al. 2013). There are also other more recent Asian records of *R. virescens* provided with molecular data, e.g. from China (Wang et al. 2022), Lao (Łuczaj et al. 2021), Myanmar (Hosaka et al. 2021) and Sri Lanka (Tibpromma et al. 2017). However, these publications used sampling covering limited geographical areas and are based mainly on ribosomal DNA regions. ITS sequences of Thai specimens in public sequence databases are often identified as *R. virescens*, but Kaewkrajang et al. (2020) reported morphological differences between Thai and European green-cracking Russulas.

Green-cracking Russulas are not only important in ethnomycology of Southeast Asia, but they are also source of interesting bioactive compounds useful in medicine that were specifically studied on material from this geographical area (Li et al. 2021). However, some *Virescentinae* are also known to have unpleasant odours that may be potentially harmful when used as food, for example *R.*

No Comments.

chlorinosma Burl. has persistent intensive odour of chlorine (Burlingham 1924). Because of this, it is particularly important to recognize correctly morphological and phylogenetic species concept within *Virescentinae*. Studies using multilocus phylogenetic analyses repeatedly confirmed that majority of *Russula* species in Southeast Asia are endemic to this area, but albeit similar to European species (Li et al. 2023). Based on dissimilarities in ITS region already reported by Cao et al. (2013), we hypothesized that there is at least one additional Asian species of green-cracking *Russula*. Our aim was to confirm if there is phylogenetic signal (using multi-locus dataset) to distinguish our recent Thai collections from European *R. virescens* and also to identify morphological differences between them in case they represent two distinct species.

Materials and methods

Studied collections and morphological observations

This study is based on five Thai collections of green-cracking Russulas (OR1607, OR1619, OR1623, OR1687 and OR1717) collected in Chiang Mai and Chiang Rai provinces. The collecting sites are represented by dry, semi-deciduous, Dipterocarpaceae-dominated forests with some Fagaceae admixture, between approximately 700 and 1050 m a.s.l. For the purpose of the study, we also included new molecular data of five European collections of *R. virescens*, in which four of them were from Slovakia and one from Belgium, two Slovak collections of *R. mustelina* Fr. and an additional collection of green-cracking *Russula* identified as *R.* cf. *viridirubrolimbata* from China. Analysed Southeast Asian specimens were dehydrated at 45°C and deposited in the herbarium of Mae Fah Luang University (MFLU, Thailand). Slovak collections are deposited in SAV herbarium. Macroscopic characters were observed on fresh material. Size, colour and features of pileus, lamellae, stipe and spore deposit were recorded. Fruiting body colours were recorded in daylight and were coded using colour charts by Kornerup and Wanscher (1967). We used the terminology for macroscopic features described and illustrated in Vellinga (1988).

We used dried materials to study microscopic characters. All samples were prepared and observed under an Olympus CX43 (Tokyo, Japan) or a Nikon Eclipse Ni (Tokyo, Japan). For the description of microscopic characters, we followed the measurement template and terminology proposed by Adamčík et al. (2019). The microscopic structures were rehydrated using aqueous 5% KOH solution and observed in Congo Red (Clémençon 1973). Spore ornamentation is described and illustrated as observed in Melzer's reagent. Line drawings were made from pictures taken using NIS Elements Software (Tokyo, Japan) or prepared using Olympus U-DA drawing attachment at a projection scale of 2000 ×. Spores were measured in side view in Melzer's reagent, excluding the ornamentation, and measurements are given as {(MIN) [AV-SD]–*AV*–[AV+SD] (MAX)}$_{length}$ × {(MIN) [AV-SD]–*AV*–[AV+SD] (MAX)}$_{width}$ in which AV = mean value for the measured collection and SD = standard deviation. Q corresponds to spore "length/width ratio" and is given as (MINQa) Qa–Qb (MAXQb), where Qa and Qb are the lowest and the highest mean ratio for the measured specimens, respectively. Lamellae and pileal tissues were treated with sulfovanillin solution (Caboň et al. 2017) to observe the colour change of cystidium contents. The colour change and incrustation of pileal tissues were checked by mounting in Cresyl blue (Buyck 1989) and carbolfuchsin (Romagnesi 1967). The statistics of all measurement were taken based on 30 measurements per collection and three collections per species were included in each species description.

Number: 1 Author: Reviewer Subject: Highlight Date: 2024-11-28 22:26:13

Hypothesis does not sound right to me. Since Cao et al. 2013 published their study, multiple species within Virescentinae were described. Additionally, there is Russula viridirubrolimbata which could potentially represent Cao´s "asian virescens". It would be beneficial better elaborate, on which basis you have assumed, that there could be higher diversity.

Number: 2 Author: Reviewer Subject: Highlight Date: 2024-11-28 22:26:23

This aim does not makes a sense to me. Previous phylogenetic studies clearly distinguished asian "virescentinae" from european. You have noted also this fact in the text above, when you state: "Soon it became apparent that at least part of so-called R. virescens in areas distant from Europe represent different species".

Therefore I do not understand, why is important to distinguish your collections from european Russula virescens rather than closely related Russula viridirubrolimbata

DNA extraction, PCR amplification and sequencing

Genomic DNA of Asian collections was extracted from fresh tissues preserved in 2% cetyl trimethylammonium bromide (CTAB) buffer, or from dried tissues, using a CTAB protocol adapted from Doyle and Doyle (1990). DNA of Slovak collections was extracted from dried basidiomata using E.Z.N.A. Fungal DNA extraction kit (Omega Bio-Tek, Norcross, Georgia) following the manufacturer's instructions. Three gene regions were used to infer phylogenetic relationships and species delimitation of studied samples: the internal transcribed spacer region (ITS) of the nuclear ribosomal DNA, the second largest subunit of RNA polymerase II (*rpb*2) and translation elongation factor1 alpha (*tef*1). The three regions were amplified using primers ITS1-F/ITS4 (White et al. 1990) for ITS, bRPB2-6F and bRPB2-7.1R (Matheny et al. 2007) or alternatively reverse primers 7cRruss1/7cRruss2 were used (Buyck et al. 2018) for *rpb*2, and EF1-983F/EF1-2218R sometimes combined with internal primers EF1-1567R and EF1-1577F (Rehner and Buckley, 2005), or EF1-983F/EF1-1567R-R (5'-GACHGTRCCRATACCACCRATYT-3'; this study) for *tef*1. The polymerase chain reaction (PCR) mix consisted of 2 ng/µl of template DNA, forward and reverse primers (10 µM), 5x HOT FIREPol® Blend Master Mix (Solis BioDyne, Estonia) and molecular grade water to bring the total volume to 20 µl. All PCR cycling conditions started with initial denaturation step at 95 °C for 15 min. For ITS was followed by 35 cycles each comprising of denaturation at 95 °C for 30 s, annealing at 56 °C for 30 s and elongation at 72 °C for 90 s., a final extension was carried out at 72 °C for 10 min. Cycling conditions for *rpb*2 were the same as described in Matheny et al. (2002) and for *tef*1 touchdown PCR procedure as described in Don et al. (1991) and Rehner and Buckley (2005).

All PCR products were checked using 1% agarose gel electrophoresis. DNA sequencing of the successful PCR products using the PCR primers were performed on an ABI 3700 automated DNA sequencer by U2Bio Thailand for Asian collections and SEQme sequencing company (Dobříš, Czech Republic) for European collections . The resulting raw sequences were manually checked, edited and assembled in Geneious R8 or R10(Biomatters).

Phylogenetic analyses

Contigs were gathered in MEGA11 (Tamura et al. 2021) or BioEdit ver. 7.2.5 (Hall 1999) and aligned using MAFFT 7.402 (Katoh 2019). Partial regions of the small subunit (SSU) and large subunit (LSU) of the nuclear ribosomal DNA were excluded from the ITS region sequences. The ITS alignment was processed with TrimAl (Capella-Gutiérrez et al. 2009), using the automated algorithm, to eliminate poorly aligned positions. *Russula floriformis* subsp. *floriformis* and *R. floriformis* subsp. *symphoniae* (subsect. *Substriatinae*) were selected as the outgroup (Vera et al. 2021). Prior to multi-locus analyses, single-gene tree topologies were compared and examined for conflicts at nodes with bootstrap support value (BS) above 70%. A combined dataset of ITS-*rpb*2-*tef*1 was assembled in BioEdit 7.2.5 (Hall 1999) and partitioned ML and BI analyses were performed. The partition comprised four character sets for the ML analysis: the ITS region, *rpb*2 exons, *tef*1 exons, and introns from *rpb*2 and *tef*1 combined. For the BI analysis, the 5.8S gene was separated from ITS1 and 2 as a fifth character set. Partitioned maximum likelihood (ML) analysis was performed in RAxML 8.2.12, with the GTRGAMMA+I model and the rapid bootstrapping algorithm for 1,000 replicates (Stamatakis 2014). The best-fit sequence evolution model was inferred for each character set using the corrected Akaike criterion in jModelest (Darriba et al. 2012). The following models were obtained: HKY + G for ITS1+ITS2, K80 for 5.8S, SYM+G for *rpb*2 exons and for *tef*1 exons, and HKY+I for introns. Partitioned BI analysis was executed in MrBayes 3.2.7a (Ronquist et al. 2012), using two runs with four chains for 2 million generations, which were sampled every 250th. Before obtaining the consensus tree from the samples, the convergence and burn-in proportion were checked using Tracer 1.6 (Rambaut et al. 2013). All phylogenetic analyses were done using the CIPRES Science Gateway (Miller et al. 2010).

No Comments.

A second, ITS-only analysis was performed with sequences gathered from public databases to gather more information about distribution of *Virescentinae* in Southeast Asia and for sequences of taxa closely related to the studied species . ITS sequences with at least 3% similarity to sequences of green-cracking Russulas obtained in this study were retrieved from UNITE database (Abarenkov et al. 2023). We also included sequences of known species of *R.* subsect. *Virescentinae* from the literature (Chen et al. 2019 and 2021, Das et al. 2017, Deng et al. 2020, Han et al. 2023, Hyde et al. 2016 and 2019, Li 2014, Paloi et al. 2023, Song et al. 2018, Ullah et al. 2020). All sequences were gathered in MEGA11 and aligned using MAFFT 7.402 (Katoh 2019). The final ITS alignment was analysed under similar conditions as the multi-locus dataset.

Table 1. Sequences used for the multi-locus analyses. Newly generated sequences are indicated in bold. Type specimens are designated with (T).

Taxa	Voucher	Country	Genbank accession number		
			***rpb*2**	**ITS**	***tef*1**
Outgroup					
R. floriformis subsp. *floriformis*	SAVF-20573 (T)	Columbia	MT021752	MT039866	MT024552
R. floriformis subsp. *symphoniae*	SAVF-20574 (T)	Panama	MT021753	MT039867	MT024553
Closely related groups in subg. *Heterophyllidiae*					
R. amoena	SAFV-3147		MT417202	MT017544	MT417211
R. bella	SFC20170731-02	South Korea	MT199645	MT017556	MT199658
R. orientipurpurea	SFC20170725-37 (T)	South Korea	MT199639	MT017548	MT199652
R. phloginea	CNX530524068 (T)	China	-	MK860701	MK894877
R. subbubalina	RITF4710 (T)	China	-	MW646978	MW650847
R. subpallidorosea	RITF4083	China	-	MK860697	MK894875
R. vesca	RITF5038	China	-	MW646984	MW650851
Subsect. *Virescentinae*					
R. aff. *virescens*	721BB-09-021	New Caledonia	KU237868	-	KU238009
R. albidogrisea	K15091234 (T)	China	-	KY767807	MN617847
R. albolutea	RITF2653 (T)	China	MW411340	MT672478	-
R. albolutea	RITF4460	China	MW411341	-	-
R. albolutea	RITF4461	China	MW411342	-	-
R. aureoviridis	RITF4709	China	MW646980	MW646980	MW650849
R. aureoviridis	H16082612 (T)	China	-	KY767809	MN617846
R. luofuensis	RITF4706	China	-	MW646973	MW650842
R. luofuensis	RITF4707	China	-	MW646974	MW650843
R. luofuensis	RITF4708 (T)	China	-	MW646975	MW650844
R. mustelina	**SAVF-2593**	**Slovakia**	**PP736577**	**PP724717**	**PP736583**
R. mustelina	**SAVF-3124**	**Slovakia**	**PP736578**	**PP724718**	**PP736584**
R. orientalovirescens sp. nov.	**OR1623**	**Thailand**	-	**PQ308639**	-
R. orientalovirescens sp. nov.	**OR1687 (T)**	**Thailand**	**PQ310350**	**PQ308640**	**PQ310348**
R. pallidula	RITF2613 (T)	China	MH091698	MH027958	MW650852

No Comments.

R. pallidula	RITF3331	China	MH091699	MH027959	MW650853
R. prolifica	18BB-06-161	Madagascar	KU237741	-	KU237890
R. pseudopunicea	BJTC-C335	China	OP156851	MW554144	-
R. pseudopunicea	BJTC-ZH1389	China	OP156852	OP133163	-
R. pseudopunicea	BJTC-ZH1392 (T)	China	OP156853	OP133164	-
R. subpunicea	RITF1435	China	MW411346	MN833637	-
R. subpunicea	RITF2648	China	MW411345	MN833638	-
R. subpunicea	RITF3715 (T)	China	MW411344	MN833635	-
R. viridirubrolimbata	**HBAU15020**	**China**	**PQ310351**	MT337527	**-**
**R. aff. *viridirubrolimbata***	**OR1717**	**Thailand**	**PQ310352**	**PQ308640**	**PQ310349**
R. virescens	**VHB38**	**Belgium**	**-**	**PQ308642**	**-**
R. virescens	**SAV F-3178**	**Slovakia**	**PP736575**	**PP724713**	**PP736579**
R. virescens	**SAV F-4207**	**Slovakia**	**-**	**PP724714**	**PP736580**
R. virescens	**SAV F-3326**	**Slovakia**	**-**	**PP724715**	**PP736581**
R. virescens	**SAV F-21192**	**Slovakia**	**PP736576**	**PP724716**	**PP736582**

Results

Phylogenetic analyses

The final multilocus alignment of ITS, *rpb*2 and *tef*1 regions consisted of 41 samples of *Russula* subgenus *Heterophillidiae* and was 1,955 bp long, including gaps, and had 799 distinct alignment patterns. All included green-cracking *Russula* collections with typical appearance of *R. virescens* originated from Asia and Europe and were placed in a single strongly supported clade (MLBS=100%). European collections of *R. virescens* formed a strongly supported (MLBS=100%) clade sister to all Asian collections of green-cracking Russulas. Asian collections were placed in two strongly supported subclades. The first Asian clade (MLBS=95%) contained three collections from Southeast China identified as *R. viridirubrolimbata*: HKAS122576 published by Wang et al. (2022) and HBAU15011 and HBAU15020 published by Deng et al. (2020). Our collections of green-cracking *Russula* OR1717 from Thailand is placed in this clade on a longer branch and might represent a distinct, closely related species. Our three other green-cracking Russulas from Thailand were placed in the second Asian clade (MLBS=92%) and are described as *R. orientalovirescens* sp. nov. in this study.

As the result of UNITE search at 3% similarity threshold, 84 collections from South East Asia were retrieved. Additional 12 sequences from USA and 12 sequences from Europe and two sequences or *R. virescens* supposedly collected in Europe with high similarity to *R. virescens* were included in the ITS analysis. General topology of ITS tree was congruent with multilocus tree, green cracking *Russula* species formed a supported clade within *Virescentinae* (MLBS=89%). Five species clusters and a singleton sequence were supported within this clade. At least one sequence of Asian origin is placed in each of these clades. The highest number of retrieved samples were placed in the clade corresponding to *R. orientalovirescens* (MLBS=79%). Among 72 samples of this clade retrieved from UNITE, 59 were from China, 8 from Laos, two from Japan and one from each Myanmar, South Korea and Thailand. Only three collections can be linked to symbiotic host plant by sequences originated from roots, OL475404 from *Picea asperata* (China), AB629011 from *Pyrola japonica* (Japan) and LC315886 from Fagaceae (Japan). Four UNITE Chinese sequences and one from Laos were placed in clade of *R. viridirubrolimbata*, together with one of our sequence from Thailand (OR1717). Two sequences from Indonesia from environmental samples associated with *Shorea* formed a distinct clade and likely represent a so far undescribed species. The singleton sequence MW374153 from

Number: 1 Author: Reviewer Subject: Highlight Date: 2024-11-28 22:26:39

In the presented tree, there is 42 samples

China possibly represent also additional species in this species complex. Two retrieved Thai sequences and one from Sri Lanka were placed in *R. virescens* clade together with mainly sequences of European origin. One Thai sequence retrieved from UNITE is placed in the clade which probably corresponds to North American *R. parvovirescens.*

Taxonomy

Russula orientalovirescens sp. nov. Wisitr. & Raspé (Fig. 3-6)

Mycobank number:

Etymology: 'oriental' means eastern and 'virescens' refers to the European *R. virescens*. The name epithet thus refers to the resemblence to *R. virescens* (by the green cracked pileipellis) and the species distribution in East and Southeast Asia.

Diagnosis: *Russula orientalovirescens* can be distinguished from similar species in subsect. *Virescentinae* by the following combination of characters: pileus surface with areolate cuticle on white background, cuticle at centre varying from pale yellow to pale green to green, cuticle near margin green to yellowish green; spores broadly ellipsoid to ellipsoid, warts frequently connected into short ridges, low isolated warts numerous; pileocystidia abundant, one-celled, relatively small; terminal cells short to long subcylindrical to subclavate, arising from ellipsoid to globose subterminal cells.

Holotype: THAILAND. Chiang Mai Province, Mae On District, Huay Kaew community forest, N18.870°-E99.292°, elev. 700 m, 6 September 2020, O. Raspé 1687 (MFLU-HT21-0044).

Pileus medium-sized, 5.3–8.7 cm [1] diam.; first hemispherical when young, later convex to plano-convex, and with a depressed centre; surface dry, often with whitish pruinose; cuticle continuous and smooth when young, turning strongly areolate in when mature, [2] especially ca. half radius towards margin; margin deflexed, striate; areolae centre [3] with pale yellow to pale green to green colours, areolae near the margin pale green to green to yellowish green, on paler white background. **Lamellae** white to yellowish white in face view, pale yellowish orange ("blanched almond") in tangential view, close, adnate-emarginate; lamellulae absent; furcations very frequent and near the stipe, edge even, concolorous. **Stipe** 4.2–6.0 × 1.0–1.9 cm, subcylindrical to cylindrical, often tapering near base; surface dry, longitudinally striate; white, turning pale brown when bruised. **Context** white, unchanging, compact to firm. **Odour** not distinctive, taste mild. **Spore print** white.

Spores (n=90) 7.4–8.1–8.8(–9.2)6.0–6.4–6.9(–7.2) [4] µm, broadly ellipsoid to ellipsoid, rarely narrowly ellipsoid, Q = (1.10–)1.18–1.26–1.32(–1.54); amyloid ornamentation of moderately distant [(3–)4–6(– [5] 7) in a 3 µm diam. circle], consist of low warts (0.3–)0.4–0.6(–0.8) µm high, occasionally fused into short chains [(0–)1–2(–3) in a 3 µm diam. circle], isolate [6] warts common, warts frequently interconnected by lower lines forming an incomplete reticulum, short ridge numerous [(0–)1–3(–5) in a 3 µm diam. circle]; suprahilar spot moderately large, inamyloid. **Basidia** (32.7–)35.5–41.6–47.7(–49.1) × (8.3–)8.7–9.7–10.6(–10.8) µm, narrowly clavate to clavate, 4-spored, partially with granular contents. **Hymenial cystidia on lamellae sides** (48.5–)52.3–65.9–79.5(–87.5) × (7.2–)7.3–8.7–10.0(–10.6) µm, widely dispersed (< 350/mm^2), narrowly fusiform to fusiform, heteromorphous, partially filled with granular and short needle-like contents, apically subacute to mucronate, occasionally slightly moniliform, appendage (1.5–)3–6(–7) µm, negative in sulfovanillin. **Lamellae edges** consist of marginal cells, basidia, basidioles, densely arranged. **Marginal cells** (21.1–)21.3–21.5–29.6 × (5.4–)5.6–6.9–8.2(–8.6) µm, cylindrical to subclavate, apically round. **Hymenial cystidia on lamellae edges** (39.3–)44.8–59.4–74.1(–81.2) × (5.1–)6.2–7.9–9.6(–9.7) µm, dispersed, emergent beyond hymenial layer, subcylindrical to narrowly fusiform, heteromorphous, granular contents, apically round to

Number: 1 Author: Reviewer Subject: Highlight Date: 2024-11-28 22:26:50

you should unify units across the manuscript. Description of R. virescens is given in mm instead of cm

Number: 2 Author: Reviewer Subject: Highlight Date: 2024-11-28 22:26:57

when mature instead of "in when"

Number: 3 Author: Reviewer Subject: Highlight Date: 2024-11-28 22:27:14

areolae in centre?

Number: 4 Author: Reviewer Subject: Highlight Date: 2024-11-28 22:27:28

7.4–8.1–8.8(–9.2) × 6.0–6.4–6.9(–7.2)

Number: 5 Author: Reviewer Subject: Highlight Date: 2024-11-28 22:31:03

Informations about ornamentation are arranged somehow chaotically. I recommend to use same order as for R. virescens

Number: 6 Author: Reviewer Subject: Highlight Date: 2024-11-28 22:30:10

isolated

mucronate, appendage up to (2.0–)2.5–5.0(–6.4) µm, negative in sulfovanillin. **Pileipellis** orthochromatic in Cresyl blue, sharply delimited from the underlying context, 75–250 µm deep, two-layered, not gelatinized, suprapellis composed of chains of short cells with attenuated hyphal termination; subpellis composed of layers of ellipsoid to globose cells. Acid-resistant incrustations absent. **Hyphal terminations near the pileus margin** ascending or erect, occasionally branched, thin-walled; terminal cells (8.6–)9.2–18.6–28.0(–33.8) × (3.8–)3.9–4.9–5.9(–6.0) µm, varying from short and broader to longer and narrower, mainly subcylindrical or broader near base, at times subclavate, apically round, subterminal cells globose to subglobose, arranged in chains, typically unbranched, (4.9–)5.7–8.9–12.1(–19.7) µm diam. **Hyphal terminations near the pileus centre** similar to those near the pileus margin; terminal cells 9.9–18.5–28.3(–37.3) × (3.3–)3.4–4.5–5.6(–6.3) µm, mainly subcylindrical, occasionally shorter and subclavate, apically round, subterminal cells globose to subglobose, arranged in chains, typically unbranched, (4.6–)5.8–7.6–9.4(–13.8) µm diam. **Pileocystidia near the pileus margin** (13.3–)14.4–20.7–26.9(–29.7) × (3.0–)3.1–3.9–4.8(–4.9) µm, small and inconspicuous, somewhat abundant, one-celled, thin-walled, erect from subterminal inflated cells, subcylindrical or tapering towards apex, partially with crystalline contents, constricted near apex, apically round. **Pileocystidia near the pileus centre** (13.6–)14.8–23.8–32.8(–37.1) × (2.7–)2.9–3.9–4.9(–5.5) µm, small and inconspicuous, abundant, similar to those near the pileus margin.

Additional examined specimens: THAILAND. Chiang Mai Province, Mae Taeng District, Baan Tha Pha, N19.141°-E98.763°, elev. 1,050 m, 9 August 2019, O. Raspé 1607 (MFLU-HT21-0042). Chiang Rai Province, Mueang District, Doi Pui community forest, N19.817°-E99.866°, elev. 720 m, 20 August 2019, O. Raspé 1619 (MFLU-HT21-0043). —, 26 August 2019, O. Raspé 1623.

Habit, habitat and distribution: Usually solitary, in dry, semi-deciduous forests dominated by Dipterocarpaceae (Dipterocarpus and Shorea spp.) in Northern Thailand. The precise distribution and ecology in neighbouring countries should be further studied.

Note: The field appearance of *R. orientalovirescens* is very similar European *R. virescens* and Chinese *R. viridirubrolimbata*, they all have greenish cracking pileus cuticle. The Chinese species is distinct in having pinkish to reddish colours near pileus margin (Ying 1983, Li 2014, Deng 2020). The European species is very similar and we were unable to define any significant macromorphological difference from the new species. However, *Russula orientalovirescens* differs from *R. virescens* in several micromorphological characters, i.e. it has larger spores, wider basidia, larger subterminal cells in pileipellis, and shorter and more numerous pileocystidia near the pileus margin. North American *R. parvovirescens* has smaller spores and more inflated subterminal cells compare to the new Asian species.

Russula virescens (Schaeff.) Fr. 1836 (Fig. 7-8)

Diagnosis: Pileus surface with distinct granules or fine areolae towards the margin, dull green to grey or dark green near the centre, context compact to firm, with indistinct and pleasant odour, spore print white; spores broadly ellipsoid to ellipsoid, subreticulate, with small, not amyloid suprahilar spot; hymenial cystidia fusiform or lanceolate, more abundant near the lamellae edges, with heteromorphous contents in almost 3/4 of the volume weakly react with sulfovanillin; pileipellis of well defined suprapellis of dessely arranged globose cells forming almost epithelium and near surface with more elongated loose terminal cells, subpellis strongly gelatinized; pileicystida inconspicuous, near mergin dispersed or absent.

Number: 1 Author: Reviewer Subject: Highlight Date: 2024-11-28 22:28:30

information about cystidioid or oleipherous hyphae is absent

Pileus medium-sized to large, 75 mm diam., first hemispherical, later convex to plano-convex, and with a slightly depressed centre, when old usually with deflexed margin; when mature indistinctly striate up to 10 mm; cuticle dry, granulose, soon cracking to small areolae that are max 5 mm large near the margin and gradually pass in ca. half radius to continuous, smooth cuticle near the centre, areolae near the margin dull green (28E4, 28D3) or greyish green (27B2) on cracked placed reveal greyish green gelatinous subpellis (27C3), areolae on sun-exposed places discolouring to greyish yellow (2B3) on paler white background (2A2), near the centre greenish grey (27E2), dull green (27E4) to dark green (27F4) and variegated with reddish blonde (5C4). **Lamellae** moderately distant, adnate-emarginate, orange white (5A2); lamellulae occasional to rare, furcations very frequent; edge irregular. **Stipe** 80 × 24 mm, cylindrical or clavate, interior solid, often narrowed near base, longitudinally strongly striate, pitted or rugulose on base white, base soon becoming yellowish white with rusty spots. **Context** white, unchanging, compact to firm, but crumbling when cut, odour indistinct, pleasant like Boletus, but in dry condition with unpleasant ammonia component. **Spore print** white (1B).

Spores (6.0–)6.6–7.2–7.8(–9.0) × (4.7–)5.3–5.7–5.9(–6.2) µm, broadly ellipsoid to ellipsoid, Q=(1.15–)1.22–1.29–1.37(–1.48); ornamentation of relatively small, moderately distant to dense [(3–)5–8(–11) in a 3 µm diam. circle] amyloid warts, (0.2–)0.4–0.6 µm high, usually fused in chains [(0–)1–4(–5) fusions in the circle] and connected by occasional to frequent line connections [(0–)1–4(–5) line connections in the circle], forming subreticulate structure, isolated warts rare or absent; suprahilar spot small, not amyloid, smooth. **Basidia** (36–)42–48.6–55.5(–60) × (6.5–)8–8.8–9.5(–10) µm, clavate, pedicellate, 4-spored; basidioles first cylindrical, then clavate, ca. 5–8 µm wide. **Hymenial cystidia** widely dispersed, ca. 130–150/mm^2, (53–)62–76.9–92(–123) × 7–8.5–9.5(–12) µm, fusiform or lanceolate, pedicellate, apically acute, usually with a 2–5 µm long appendage, thin-walled; contents heteromorphous in almost 1/2 to 3/4 of the volume, granulose-crystalline to banded, apically more dense, almost negative in sulfovanillin; near the lamellae edges more frequent, smaller, (33–)44.5–56.4–68.5(–78) × (5–)5.5–6.8–8(–9) µm, narrowly fusiform, clavate, lanceolate, subcylindrical, apically acute or obtuse, without or with 2–10(–13) µm long appendage; contents usually optically empty, or granulous only in terminal 1/4 part. Lamellae edges sterile; **marginal cells** (14–)20–26.8–34(–44) × (4–)5–5.7–6.5(–8) µm, thin walled, fusiform, cylindrical, clavate, often flexuous, apically obtuse or constricted, rarely also with small outgrowths or nodulose. **Pileipellis** orthochromatic in Cresyl Blue, not sharply delimited from the underlying context, 250–330 µm thick, strongly gelatinized throughout; well divided in 55–110 µm thick, not gelatinised suprapellis of subglobose, densely arranged cells forming epithelium and near surface of ascending or erect, more loose hyphal terminations; and in a well-defined, 200–280 µm deep, strongly gelatinised subpellis of loose and irregularly oriented but near trama more dense and horizontally oriented, intricate, 2–3.5(–4) µm wide hyphae. **Acid-resistant incrustations** absent. **Hyphal terminations near the pileus margin** composed of 1–3(–5) cells until they join a basal branched cells, thin-walled; terminal cells (5–)7.5–22.9–38(–82) × (3–)4–5.6–7.5(–13) µm, mainly subulate and apically obtuse or attenuated, occasionally ellipsoid or clavate, and apically obtuse; subterminal cells usually unbranched, shorter, ellipsoid or subglobose, (4.5–)5.5–9.7–13.5(–25) × (2.5–)3.5–6.2–8(–10) µm. **Hyphal terminations near the pileus centre** similar, terminal cells (5–)10.5–23.1–35.5(–59) × (2.5–)3.5–5.3–7(–10.5) µm. **Pileocystidia near the pileus margin** dispersed, one-celled, narrowly cylindrical or subcylindrical, not distinctly narrowing towards the bases, thin-walled, (21–)31–46.7–62(–107) × 2.5–3.3–4(–4.5) µm, apically mainly obtuse and with 2–6 µm long appendage, contents optically empty or only with few dispersed granulations or crystals near terminal part, almost negative in sulfovanillin. **Pileocystidia near the pileus centre** similar, more frequent, (18.7–)25–42.7–60.5(–87) × (2–)2.5–3.5–4(–5) µm, near apical part often moniliform. Cystidioid and oleipherous hyphae in subpellis and context not observed.

No Comments.

Material studied: Slovakia, Tríbeč Mts., E of the village Podhorany, temperate forest, on ground under *Quercus*, *Carpinus* and *Fagus*, coord. 48°22'28"N, 18°08'42"E, 7.9.2022, M. Adamčík jun., SAV F-21192 (S948); Tríbeč Mts., Jelenec, N of the autocamp, temperate forest, on ground under *Quercus* and *Carpinus*, coord. 48°24'32"N, 18°12'8"E, 12.7.2010, S. Adamčík, SAV F-3178 (S886); Štiavnické vrchy Mts., Sebechleby, Stará hora, temperate forest, on ground under Quercus and Carpinus, coord. 48°16'54"N, 18°54'13"E, 13.7.2010, S. Adamčík, SAV F-4207 (S887); Krupinská planina Mts., Plášťovce, NE of Krašoria hill, temperate forest, on ground under *Quercus* and *Carpinus*, coord. 48°11'55.46"N, 19° 3'17.48"E, 4.7.2011, T. Christiansen, SAV F-3326 (S888).

Note: The *Virescentinae* were for a long period defined as a group without pileocystidia and with exclusively areaolate pileus cuticle (Singer 1986). Some morphological classification concepts either expanded concept of *Virescentinae* to group all *Russula* species of subgenus *Heterophyllidia* without pileocystidia (Romagnesi 1967) or raised status of areolate species to a section rank (Sarnari 1998). Pileocystidia in *R. virescens* were first time observed by Buyck (2004). Pileocystidia of this species were overlooked for a long time, because they are very dispersed and inconspicuous, and almost absent near the pileus margin. Here we confirmed the presence of pileocystidia in the type species of subsection *Virescentinae*, *R. virescens*. Buyck and Adamčík (2011) described presence of pileocystidia also in other *Virescentinae* members with areolate pileus in North America.

Discussion

The green-cracking Russulas *R. orientalovirescens*, *R. parvovirescens* *R. virescens* and *R. viridirubrolimbata* form a monophyletic group characterized morphologically by their distinctly areolate pileus margin combined with dominant green, grey and yellow colours. The only species in this group which exhibit also red colours near pileus margin is *R. viridirubrolimbata*, which seems to be easily recognizable from the other three species (Ying 1983). *Russula parvovirescens* is a North American species recognized by smaller size of basidiomata and more inflated (15-25 µm) subterminal cells in the pileipellis compare to *R. virescens* and *R. orientalovirescens* (Buyck et al. 2006). Unlike the older European (Romagnesi 1967, Sarnari 1998) and Chinese literature (Deng 2020), we confirmed that both Asian new species and European *R. virescens* have pileocystidia.

Our phylogenetic analysis of ITS region confirmed that *R. orientalovirescens* was previously reported as *R. virescens* in Southeast Asia. Collections of this Asian species reported as *R. virescens* are represented by 42 sequences published by Cao et al. (2013) from China, another two sequences from China published by Wang et al. (2022), four sequences from Laos published by Łuczaj et al. (2021) and one sequence from Myanmar (Hosaka et al. 2021). Deng et al. (2020) recognized eight sequences of the newly described species from China as *R.* aff. *virescens* and they treated it as different from the European species by shorter hyphal extremities in pileipellis. Three publicly available sequences originating from Thailand and Sri Lanka and identified as *R. virescens* grouped in *R. virescens* species clade and one Thai collection identified as the same species is placed in *R. parvovirescens* clade. We were unable to access Thai material or DNA corresponding to these sequences, but we doubt that both North American and European species occur in subtropical or tropical areas of Southeast Asia. It is possible, that publicly available sequences were used as template to edit low quality sequences, or there are more species with very little difference in ITS. The sequence KY649467, originating from Sri Lanka, was published as *R. virescens* and clustered in our ITS tree with European samples of this species, but it looks very different from European species on the photo, its pileipellis is not areolate, basidiomata are thin-fleshed and smaller, pileus is yellow-green and has bright orange spots near the pileus centre (Tibpromma et al. 2017). This indicate that Asian green-cracking Russulas require more

No Comments.

detailed study using multilocus phylogenetic analysis and detailed morphological observations to distinguish them from their relatives.

Similar but according to ITS tree unrelated to the new species described here is *R. pseudocrustosa* G.J. Li & C.Y. Deng which also has areolate green pileus surface, but differs by having yellowish brown to reddish brown pileus colours, cream spore deposit and lower warts (0.2–0.5 µm high) on spores (Deng et al. 2020). *Russula sribuabanensis* has also pruinose green pileus but without distinct areolae, it differs also by low reticulate spore ornamentation, larger hymenial cystidia (61–109 × 8–14 µm) and rare or absent pileocystidia (Paloi et al. 2023).

Russula species often receive good reputation for their culinary value due to their texture and flavour after cooking. In southern China and Southeast Asia, wild edible mushrooms are commonly gathered from the forests during rainy season (June to October) and sold in open air markets close to the collecting sites. Green-cracking Russulas are well-known as one of most valuable edible mushrooms and are widely consumed in southern China, Laos and Thailand. They are commonly known as Hed Lom Kra Khiao in Thai, Koh Ket Khiew or Khai Thao in Laos and Qingtoujun in Chinese (Jones et al. 1994, Sanmee et. al. 2003, Chandrasrikul 2008, Dell et al. 2011, Læssøe et al. 2018, Deng et al. 2020). In Laos, at least two forms were recorded; *R.* cf. *virescens* and *R.* cf. *viridirubrolimbata* (Læssøe et al. 2018), the first one probably corresponding to *R. orientalovirescens*. In Thailand, green-cracking Russulas are named as *R. virescens* and often are highly priced in northern provinces (Jones et al. 1994, Sanmee et al. 2003). Local people often cook the mushroom by boiling, steaming or grilling and mix them with chili paste or use for folk remedies related to cancer prevention. In China, green-cracking Russulas are among the most popular wild edible mushrooms in Yunnan province, southwestern China (Cao et al. 2013). Another Chinese ethnomycological study reported that fresh and dry *R. virescens* are sold by ethnic groups in southwestern Yunnan, China. The mushroom was frequently stir-fried with garlic and fresh chili or cooked with meat soup (Wang et al. 2022). In addition, the mushroom has been reported to contain carbohydrates, proteins, minerals and have a low glycemic index (GI) which is suitable for people who wish to control their blood sugar (Sanmee et al. 2003, On-nom et al. 2023). Another recent study reported that the Chinese *R. virescens* samples possess polysaccharides that have potential for inhibiting cancer cell proliferation and activating immune response (Li et al. 2021). Because of its culinary and medicinal values, we believe that the detailed description of our new species and comparisons with similar species can improve knowledge on the identification of this edible species and related species in the region. Our phylogenetic, morphological and ecological observations suggest, that *R. orientalovirescens* is the most common popular edible fungus occurring in various subtropical deciduous and coniferous forests with dry season and distributed from Malay Peninsula, south China, Korean Peninsula to Japan.

Page:18

Number: 1 Author: Reviewer Subject: Highlight Date: 2024-11-28 22:28:44

superfluous

Table 2. Comparisons of microscopic characters of *R. virescens* and *R. orientalovirescens*. The measurement units of all characters except Q value are µm. Characters showing distinct differences are highlighted

**Characters**	***R. virescens***			***R. orientalovirescens***		
	**SAV F-3178**	**SAV F-3326**	**SAV F-21192**	**OR1607**	**OR1619**	**OR1687**
Spores	6.4–7.2 × 5.3–5.8	7.3–8.2 × 5.5–6.0	6.6–7.4 × 5.1–5.7	7.5–8.6 × 6.1–6.7	7.4–8.2 × 6.0–6.6	7.6–9.2 × 6.1–7.2
Q value	1.18–1.27	1.29–1.41	1.24–1.36	1.18–1.33	1.18–1.29	1.19–1.34
Basidia	43.6–51.9 × 8.4–9.2	38.0–46.0 × 7.9–9.3	52.7–59.4 × 8.4–9.4	42.6–49.1 × 9.4–10.8	37.3–46.9 × 9.4–10.7	32.7–41.0 × 8.3–9.5
Marginal cells	16.9–27.1 × 5.2–7.6	23.4–37.8 × 4.4–6.0	22.2–33.5 × 4.9–6.4	22.4–29.4 × 5.4–7.2	21.1–29.0 × 6.4–8.6	21.7–29.4 × 5.8–8.0
Pleurocystidia	74.2–102.0 × 7.2–9.8	57.7–80.6 × 7.1–8.5	60.2–78.4 × 7.4–10.0	68.7–87.5 × 7.4–9.3	48.5–67.2 × 7.2–9.6	54.4–69.3 × 8.0–10.6
Cheilocystidia	53.8–75.9 × 6.3–7.7	38.8–55.0 × 5.3–7.7	48.5–66.8 × 5.8–8.1	54.3–81.2 × 7.0–9.3	39.3–62.4 × 5.1–8.7	51.0–68.4 × 7.7–9.7
Terminal cells margin	5.9–22.5 × 4.7–7.5	18.1–53.7 × 3.9–8.3	11.4–25.9 × 3.9–5.4	17.5–33.8 × 3.9–5.8	8.6–13.1 × 4.1–5.8	13.0–25.6 × 3.8–6.0
Pileocystidia margin	30.6–60.7 × 3.2–4.1	32.8–56.4 × 2.9–3.8	29.7–68.6 × 2.7–3.7	16.7–29.7 × 3.0–4.9	13.3–24.1 × 3.2–4.4	16.2–24.1 × 3.4–4.8
Terminal cells centre	5.7–28.1 × 4.8–8.2	16.3–43.7 × 3.3–4.9	14.2–31.2 × 4.0–6.3	16.8–37.3 × 4.3–6.3	11.9–18.4 × 3.3–4.6	9.9–16.8 × 3.5–5.1
Pileocystidia centre	28.3–68.6 × 3.3–4.2	28.0–64.7 × 3.0–4.8	23.6–45.3 × 2.3–3.6	17.5–37.1 × 3.3–5.5	13.6–22.0 × 3.5–4.1	20.5–32.0 × 2.7–4.3

Number: 1 Author: Reviewer Subject: Highlight Date: 2024-11-28 22:28:53

I suggest to add also comparisons with the other species - R. parvovirescens and R. viridirubrolimbata, from literature

Number: 2 Author: Reviewer Subject: Highlight Date: 2024-11-28 22:29:03

In the description, you are refferring to pleurocystidia as hymenial cystidia on lamellae sided / cheilocystidia on lamellae edges

Authors' contribution:

KW, BC, OR and SA collected materials and prepared morphological descriptions, KW, OR and SA wrote the manuscript, KA, OR and ST performed molecular analyses and submitted the sequences to GenBank, OR and KW constructed the phylogenetic trees, all authors commented on and agreed with the text.

Acknowledgements

This work was financially supported by Mae Fah Luang University grant 641A01003, "Survey of edible fungi in dry dipterocarp forests of Chiang Mai Province, Thailand" to O. Raspé. Komsit Wisitrassameewong was supported by the Ecological Monitoring and Plant Specimen and Barcode References project P2250745, National Science and Technology Development Agency. The work of Slovak authors was supported by the Slovak Research and Development Agency projects APVV-15-0210 and APVV-20-0257.

References

- Abarenkov K., Nilsson R.H., Larsson K.-H., Taylor A.F.S., May T.W., Frøslev T.G., Pawlowska J., Lindahl B., Põldmaa K., Troung C. et al. (2023). The UNITE database for molecular identification and taxonomic communication of fungi and other eukaryotes: sequences, taxa and classifications reconsidered. Nucleic Acids Research 52(D1): D791-D797. http://doi.org/10.1093/nar/gkad1039.
- Adamčík S., Looney B., Caboň M., Jančovičová S., Adamčíkova K., Avis P.G., Barajas M., Bhatt R.P., Correles A., Das K., et al. (2019). The quest for a globally comprehensible *Russula* language. *Fungal Diversity* 99: 369–449.
- Altschul S.F., Gish W., Miller W., Myers E.W., Lipman D.J. (1990). Basic local alignment search tool. Journal of Molecular Biology 215(3): 403-410. Doi:10.1016/S0022-2836(05)80360-2.
- Anh C.N., Chi N.M., Kiet T.T., Long P.D., Thuy P.T.T., Loi V.V., Dell B. (2023). Morphological and molecular identification of an edible *Russula* mushroom in northern Vietnam. Journal of Forestry Science and Technology 15: 50–59.
- Beardslee H.C. (1918). The Russulas of North Carolina. Kessinger Publishing. ISBN-10: 1167190572.
- Burlingham G.S. (1915). *Russula*. *North American Flora* 9: 201–236.
- Burlingham G.S. (1924). Notes on species of *Russula*. *Mycologia* 16: 16–23.
- Buyck B. (1989). Valeur taxonomique du bleu de crésyl pour le genre *Russula*. *Bulletin trimestriel de la Société Mycologique de France* 105: 1–6.
- Buyck 2004: Précisions sur *Russula virescens* et sur quelques *Griseinae* leucosporées. *Bulletin trimestriel de la Société mycologique de France* 120: 385-401.
- Buyck B., Parrent J., Vilgalys R. (2005). The *Russula virescens-crustosa* species complex (Russulales, Basidiomycota) from eastern North America. Inoculum 56(4): 11.
- Buyck B. & Adamčík S. (2011). Type studies in *Russula* subgenus *Heterophyllidia* from the eastern United States. *Cryptogamie Mycologie* 32(2): 151–169.
- Buyck B., Zoller S., Hofstetter V. (2018). Walking the thin line... ten years later: The dilemma of above- versus below-ground features to support phylogenies in the Russulaceae. *Fungal Diversity* 89: 267–292.
- Caboň M., Eberhardt U., Looney B., Hampe F., Kolařík M., Jančovičová S., Verbeken A., Adamčík S. (2017). New insights in *Russula* subsect. *Rubrinae*: phylogeny and the quest for synapomorphic characters. *Mycological Progress* 16: 877–892.

No Comments.

- Cao Y., Zhang Y., Yu Z., Mi F., Liu C., Tang X., Long Y., He X., Wang P., Xu J. (2013). Structure, gene flow, and recombination among geographic populations of a *Russula virescens* ally from Southwestern China. *PLoS One* 8(9): e73174.
- Capella-Gutiérrez S., Silla-Martínez J.M. & Gabaldón T. (2009) TrimAl: a tool for automated alignment trimming in large-scale phylogenetic analyses. *Bioinformatics* 25: 1972–1973
- Chandrasrikul A. (2008). Diversity of Mushrooms and Macrofungi in Thailand. Kasetsart University Press.
- Chen B., Jiang X., Song J., Liang J., Wang S., Lu J. (2019). Morphological and phylogenetic analyses reveal the new species *Russula pallidula* from China. *Sydowia* 71: 1–10. 10.12905/0380.sydowia71-2019-0001.
- Chen B., Song J., Zhang J., Liang J. (2021). Morphological and phylogenetic evidence for two new species of *Russula* subg. *Heterophyllidia* from Guangdong province of China. *Mycokeys* 82: 139–157.
- Clémençon H. (1972). Zwei verbesserte Präparierlösungen für die microskopische Untersuchung von Pilzen. *Z. Pilzk* 38, 49–53.
- Corrales, A., Henkel, T.W. and Smith, M.E. (2018), Ectomycorrhizal associations in the tropics – biogeography, diversity patterns and ecosystem roles. New Phytol, 220: 1076-1091. https://doi.org/10.1111/nph.15151
- Darriba D., Taboada G.L., Doallo R., & Posada D. (2012) jModelTest2: more models, new heuristics and high-performance computing. *Nature Methods* 9: 772. https://doi.org/10.1038/nmeth.210
- Das K., Ghosh A., Chakraborty D., Li J., Qiu L., Baghela A., Halama M., Hembrom M., Mehmood T., Parihar A., Pencakowski B., Bielecka M., Reczyńska K., Sasiela D., Singh U., Song Y., Świerkosz K., Szcześniak K., Uniyal P., Zhang J., Buyck B. (2017). Fungal Biodiversity Profile 31-40. *Cryptogamie Mycologie* 38(3): 353–406.
- Deng C.Y., Lu-Yao S., Jing W., Zhun X., Shou-Mian L., Guo-Jie L., Hui Y. (2020). Species diversity of the *Russula virescens* complex "qingtoujun" in southern China. *Mycosystema* 39(9): 1661–1683.
- Don R.H., Cox P.T., Wainwright B.J., Baker K., Mattick J.S. (1991). Touchdown PCR to circumvent spurious priming during gene amplification. *Nucleic Acids Research* 19: 4008.
- Doyle J.J., Doyle J.L. (1990) Isolation of plant DNA from fresh tissue. *Focus* 12:13–15.
- Gillet C.C. (1876). Les Hyménomycètes ou Description de tous les Champignons qui Croissent en France.
- Gómez P.L.D., Alfaro R.M. (1996). Basidiomicetes de Costa Rica. De Russulae novae. *Revista de Biologia Tropical* 44: 25–37.
- Hall T.A. (1999). BioEdit: A user-friendly biological sequence alignment editor and analysis program for windows 95/98/NT. *Nucleic Acids Symposium Series* 41: 95–98.
- Han Y.X., Liang Z.Q., Zeng N.K. (2023). Notes on four species of *Russula* subgenus *Heterophyllidiae* (Russulaceae, Russulales) from southern China. *Frontiers in Microbiology*. Doi:10.3389/fmicb.2023.1140127.
- Hongo T. (1979). Notulae Mycologicae 16. *Memoirs of Shiga University* 29: 99–104.
- Hosaka K., Nam K.O., Linn W.W., Aung M.M. (2021). Species identification based on DNA of selected mushrooms from Myanmar (1) *Lactarius austrotorminosus* and 17 other taxa newly reported from Myanmar. *Bulletin of the National Museum of Nature and Science, Series B*. 47(2): 59–69.
- Hyde K.D., Hongsanan S., Jeewon R., Bhat D.J., Mckenzie E.H.C., Gareth Jones E.B., Phookamsak R., Ariyawansa H.A., Boonmee S., Zhao Q. et. al. (2016). Fungal diversity notes 367-490: taxonomic and phylogenetic contributions to fungal taxa. *Fungal Diversity* 80: 1–270.

No Comments.

- Hyde K.D., Tennakoon D.S., Jeewon R., Bhat D.J., Maharachchikumbura S.S.N., Rossi W., Leonardi M., Lee H.B., Mun H.Y., Houbraken J. et. al. (2019). Fungal diversity notes 1036-1150: taxonomic and phylogenetic contributions on genera and species of fungal taxa. *Fungal Diversity* 96: 1–242.
- Kaewkrajang T., Kaewjunsri S., Jannual N., Nipitwattanaphon M. (2020). Morphological and molecular identification of some *Lactarius* and *Russula* species. *Genome and Genetics* 13(2): 44–58.
- Katoh K., Rozewicki J., Yamada K.D. (2019). MAFFT online service: Multiple sequence alignment interactive sequence choice and visualization. *Briefings in Bioinformatics* 20(4): 1160–1166. http://doi.org/10.1093/bib/bbx108.
- Kearse M., Moir R., Wilson A., Stones-Havas S., Cheung M., Sturrock S., Buxton S., Cooper A., Markowitz S., Duran C., et al. (2012). Geneious basic: an integrated and extendable desktop software platform for the organization and analysis of sequence data. *Bioinformatics* 28: 1647–1649. doi:10.1093/ bioinformatics/bts199.
- Kornerup A, Wanscher JH. 1967. Methuen handbook of colour. 2nd ed. London: Methuen Co.
- Li G.J. (2014). The taxonomy of *Russula* in China. PhD dissertation. University of Chinese Academy of Sciences, China.
- Li G.J., Li S.M., Buyck B., Zhao S.Y., Xie X.J., Shi L.Y., Deng C.Y., Meng Q.F., Sun Q.B., Yan J.Q., Wang J., Li M. (2021). Three new *Russula* species in sect. *Ingratae* (Russulales, Basidiomycota) from southern China. *Mycokeys* 84: 103–139.
- Li G.J., Liu T.Z., Li S.M., Zhao S.Y., Niu C.Y., Liu Z.Z., Xie X.J., Zhang X., Shi L.Y., Guo Y.B. et. al. (2023). Four new species of *Russula* subsection *Sardoninae* from China. *Journal of Fungi* 9: 199. http://doi.org/10.3390.jof9020199.
- Łuczaj L., Lamxay V., Tongchan K., Xayphakatsa K., Phimmakong K., Radavanh S., Kanyasone V., Pietras M., Karbarz M. (2021). Wild food plants and fungi soil in the markets of Luang Prabang, Lao PDR. *Journal of Ethnobiology and Ethnomedicine* 17: 6. http://doi.org/10.1186/s13002-020-00423-y.
- Manassila M., Sooksa-Nguan T., Boonkerd N., Rodtong S., Teaumroon N. (2005). Phylogenetic diversity of wild edible *Russula* from Northeastern Thailand on the basis of the internal transcribed spacer sequence. *ScienceAsia* 31: 323.
- Matheny P.B., Liu Y.J., Ammirati J.F., Hall B.D. (2002). Using RPB1 sequences to improve phylogenetic inference among mushrooms (*Inocybe*, Agaricales). *American Journal of Botany* 89: 688–698.
- Matheny P.B., Wang Z., Binder M., Curtis J.M., Lim Y.W., Henrik Nilsson R., Hughes K.W. Hofstetter V., Ammirati J.F., Schoch C.L. et. al. (2007). Contributions of *rpb2* and *tef1* to the phylogeny of mushrooms and allies. *Molecular Phylogenetics and Evolution* 43: 430–451.
- Miller M.A., Pfeiffer W., Schwartz T. (2010). Creating the CIPRES science gateway for inference of large phylogenetic trees. In Proceeding of the Gateway Computing Environments Workshop (GCE) 1–8. https://doi. org/10. 1109/GCE.2010.5676129
- Morgan A.P. (1883). The mycological flora of Miami Valley, Ohio. *The Journal of the Cincinnati Society of Natural History* 6: 173–199.
- Murrill W.A. (1916). Prunulus. *North American Flora* 9: 319–344.
- Læssøe T., Pedersen O.S., Sysouphanthong P. (2018). An introduction to the edible, poisonous and medicinal fungi of Noerthern Laos.
- Paloi S., Kumla J., Karunarathna S.C., Lumyong S., Suwannarach N. (2023). Taxonomic and phylogenetic evidence reveal two new *Russula* species (*Russulaceae, Russulales*) from northern Thailand. *Mycological Progress* 22: 72.
- Peck C.H. (1872). Report of the Botanist (1870). Annual report on the New York State Museum of Natural History. 24: 41–108.

No Comments.

- Rambaut A., Suchard M.A., Xie D., Drummond A.J. (2014). Tracer 1.6. Available from: http://beast.bio.ed.ac.uk.
- Rehner S.A., Buckley E. (2005). A *Beauveria* phylogeny inferred from nuclear ITS and EF1- α sequences: evidence for cryptic diversification and links to *Cordyceps* teleomorphs. *Mycologia* 97(1): 84–98. doi: 10.1080/15572536.2006.11832842.
- Romagnesi H. (1967). Les Russules d'Europe et d'Afrique du Nord. ISBN: 978-3768213165.
- Ronquist F., Teslenko M., van der Mark P., Ayres D.L., Darling A., Höhna S., Larget B., Liu L., Suchard M.A., Huelsenbeck J.P. (2012). MrBayes 3.2: efficient Bayesian phylogenetic inference and model choice across a large model space. *Systematics Biology* 61(3): 539-542. Doi:10.1093/sysbio/sys029.
- Sanmee R., Dell B., Lumyong P., Izumori K., Lumyong S. (2003). Nutritive value of popular wild edible mushrooms from northern Thailand. Food Chemistry 82(4): 527-532. https://doi.org/10.1016/S0308-8146(02)00595-2.
- Sarnari M. (1998). Monographia Illustrata del Genere Russula in Europa (Associazioni Micologica Bresadola).
- Shaffer R.L. (1970). Cuticular terminology in *Russula* (Agaricales). *Brittonia* 22(3): 230–239.
- Singer R. (1932). Monographie der Gattung *Russula*. *Beihefte zum Botanischen Centralblatt* 49: 250–380.
- Singer R. (1986). The Agaricales in Modern Taxonomy. 4th edn. Koeltz Scientific books, Koenigstein.
- Song Y., Li J., Buyck B., Zheng J., Qiu L. (2018). *Russula verrucospora* sp. nov. and *R. Xanthovirens* sp. nov., two novel species of *Russula* (Russulaceae) from southern China. *Cryptogamie Mycologie* 39(1): 129–142.
- Stamatakis A. (2014). RAxML version 8: A tool for phylogenetic analysis and post-analysis of large phylogenies. *Bioinformatics* 30: 1312–1313. htttp://doi.org/10.1093/bioinformatics/btu033.
- Tamura K., Stecher G., Kumar S. (2021). MEGA11: Molecular Evolutionary Genetics Analysis Version 11. *Molecular Biology and Evolution* 38(7): 3022–3027.
- Tibpromma S., Hyde K.D., Jeewon R., Maharacchikumbura S.S.N., Liu J.K., Bhat D.J., Gareth Jones E.B., Mckenzie E.H.C., Camporasi E., Bulgakov T.S., Doilom M. et. al. (2017). Fungal diversity notes 491-602: taxonomic and phylogenetic contributions to fungal taxa. *Fungal Diversity* 83: 1–261.
- Ullah S., Wilson A.W., Fiaz M., Hussain S., Mueller G.M., Khalid A.N. (2020). *Russula shanglaensis* sp. nov. (Basidiomycota: Russulales), a new species from the mixed coniferous forests in district Shangla, Pakistan. *Turkish Journal of Botany* 44: 85–92.
- Vellinga E.C. (1988). Glossary. In *Flora Agaricina Neerlandica* vol.1 (eds Bas C., Kuyper T.W., Noordeloos M.E., Vellinga E.C.) 54–55. ISBN:9789061918592.
- Vera M., Adamčík S., Adamčíková K., Hampe F., Caboň M., Manz C., Ovrebo C., Piepenbring M., Corrales A. (2021). Morphological and genetic diversification of *Russula floriformis*, sp. nov., along the Isthmus of Panama. *Mycologia*. 113 (4): 807–827. doi:10.1080/00275514.2021.1897377. PMID: 34043494.
- Wang X.H., 2020. Taxonomic comments on edible species of Russulaceae. *Mycosystema*, 39(9): 1617-1639.
- Wang R., Herrera M., Xu W., Zhang P., Moreno J.P., Colinas C., Yu F. (2022). Ethnomycological study on wild mushrooms in Pu'er Prefecture, Southwest Yunnan, China. *Journal of Ethnobiology and Ethnomedicine* 18(55): 1–24. http://doi.org/10.1186/s13002-022-00551-7.
- White T.J., Bruns T., Lee S., Taylor J. (1990). Amplification and direct sequencing of fungal ribosomal RNA genes for phylogenetics. In PCR protocols: A guide to methods and applications (eds Innis M.A. et al.) 315–322. http://doi.org/10.1016/B978-0-12-372180-8.50042-1.
- Ying J.Z. 1983. A study on *Russula viridi-rubrolimbata* sp. nov. and its related species of subsection Virescentinas. Acta Mycologica Sinica 2(1): 34-37.

No Comments.

Figures

Figure 1. Maximum Likelihood (ML) phylogeny based on internal transcribed spacer (ITS) sequences of most *R.* subsect. *Virescentinae* and closely related species. Species in boldface are generated in this study. ML bootstrap values >70 and Bayesian Inference posterior probability >0.9 are shown. Asterisks indicate clades with 100 ML bootstrap values and 1.0 Bayesian posterior probabilities.

Figure 2. Maximum Likelihood (ML) phylogeny based on internal transcribed spacer (ITS), the second largest subunit of RNA polymerase II (*rpb2*) and the translation elongation factor 1-alpha (*tef1*) sequences of *R.* subsect. *Virescentinae* and closely related species. Species in boldface are generated in this study. ML bootstrap values >70 and Bayesian Inference posterior probability >0.9 are shown. Asterisks indicate clades with 100 ML bootstrap values and 1.0 Bayesian posterior probabilities.

Figure 3. Basidiomata of described species. A–C: *R. orientalovirescens* (A: OR1607, B: OR1619, C: OR1687), D: *R. virescens* (SAV F-21192). Photographs: A–C by O. Raspé, D by S. Adamčík.

Figure 4. Hymenial elements of *R. orientalovirescens* (OR1687). A. Basidiospore, B. Basidia, C. Marginal cell, D. Hymenial cystidia on lamellae side, E. Hymenial cystidia on lamellae edge. Illustration by K. Wisitrassameewong. Scale bar = 10 µm.

Figure 5. Elements in pileus cuticle of *R. orientalovirescens* (OR1687). A. Pileocystidia on pileus centre, B. Hyphal termination on pileus centre, C. Pileocystidia on pileus margin, D. Hyphal termination on pileus margin. Illustration by K. Wisitrassameewong. Scale bar = 10 µm.

Figure 6: SEM pictures of *R. orientalovirescens* spores. A: OR1607, B: OR1619, C: OR1687. Photographs by B. Chuankid. Scale bar = 5 µm.

Figure 7. Hymenial elements of *R. virescens* (SAV-F 3978). A. Basidiospore, B. Basidia, C. Marginal cell, D. Hymenial cystidia on lamellae edge, E. Hymenial cystidia on lamellae side. Illustration by S. Adamčík. Scale bar = 10 µm.

Figure 8. Elements in pileus cuticle of *R. virescens* (SAV-F 3978). A. Pileocystidia on pileus centre, B. Hyphal termination on pileus centre, C. Pileocystidia on pileus margin, D. Hyphal termination on pileus margin. Illustration by S. Adamčík. Scale bar = 10 µm.

No Comments.

Figure1

Click here to access/download;Figure;Fig 1. ITS phylogeny resized.tif

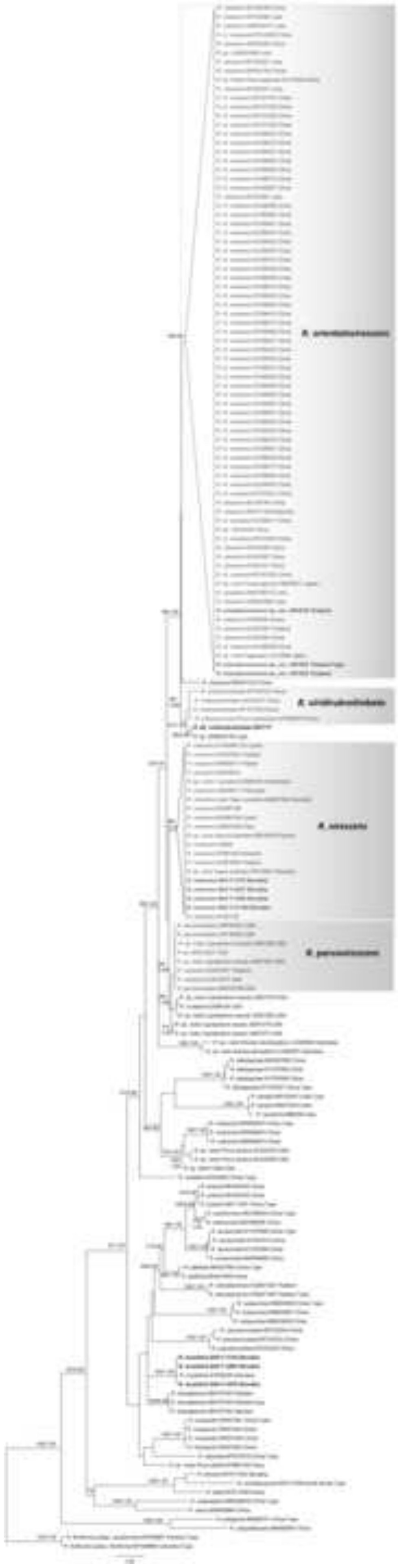

No Comments.

Figure2

Click here to access/download;Figure;Fig 2. ITS-rpb2-tef1 tree.tif

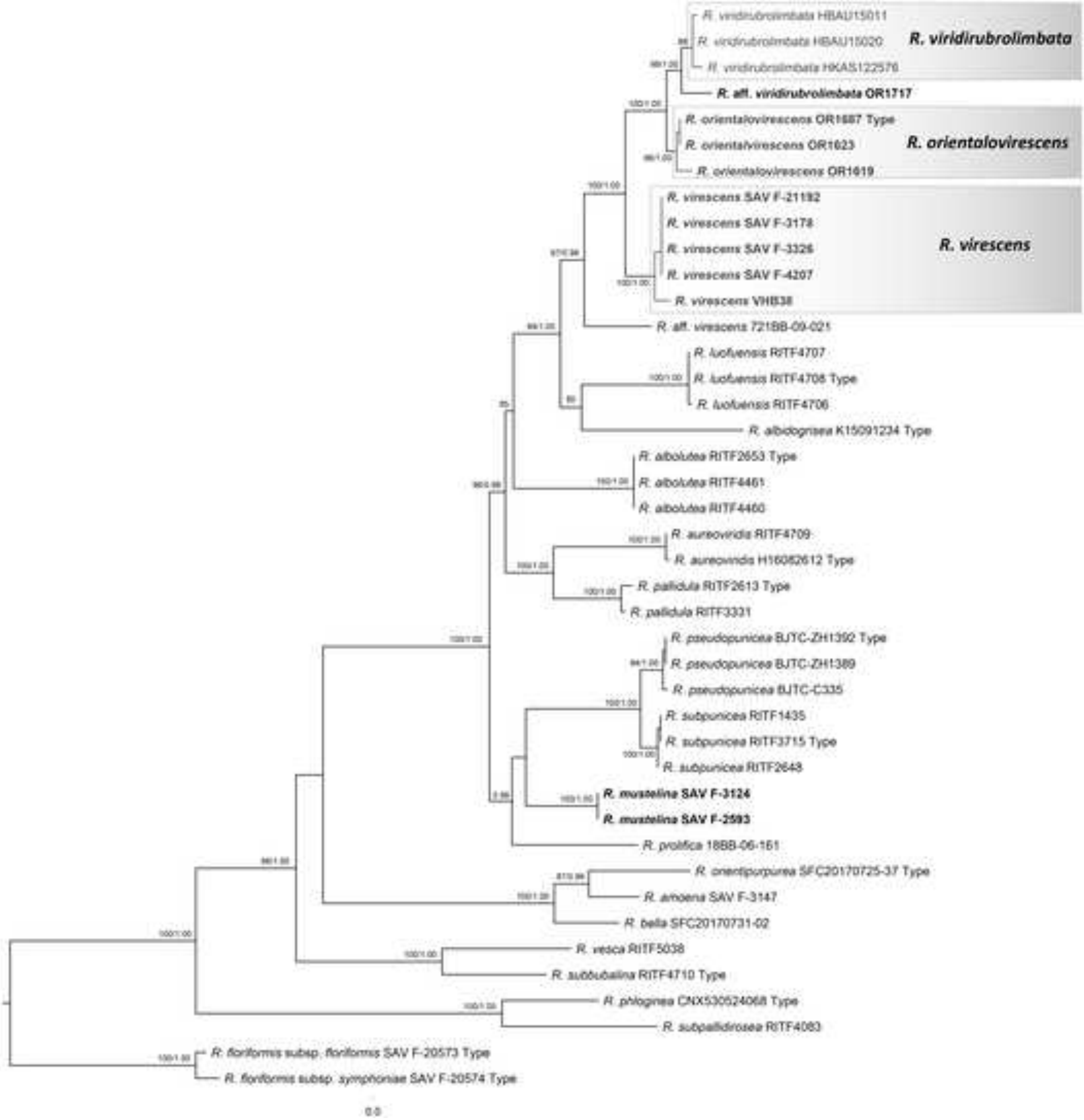

No Comments.

Figure3

Click here to access/download;Figure;Fig 3. Fruiting body R. orientalovirescens and R. virescens.tif

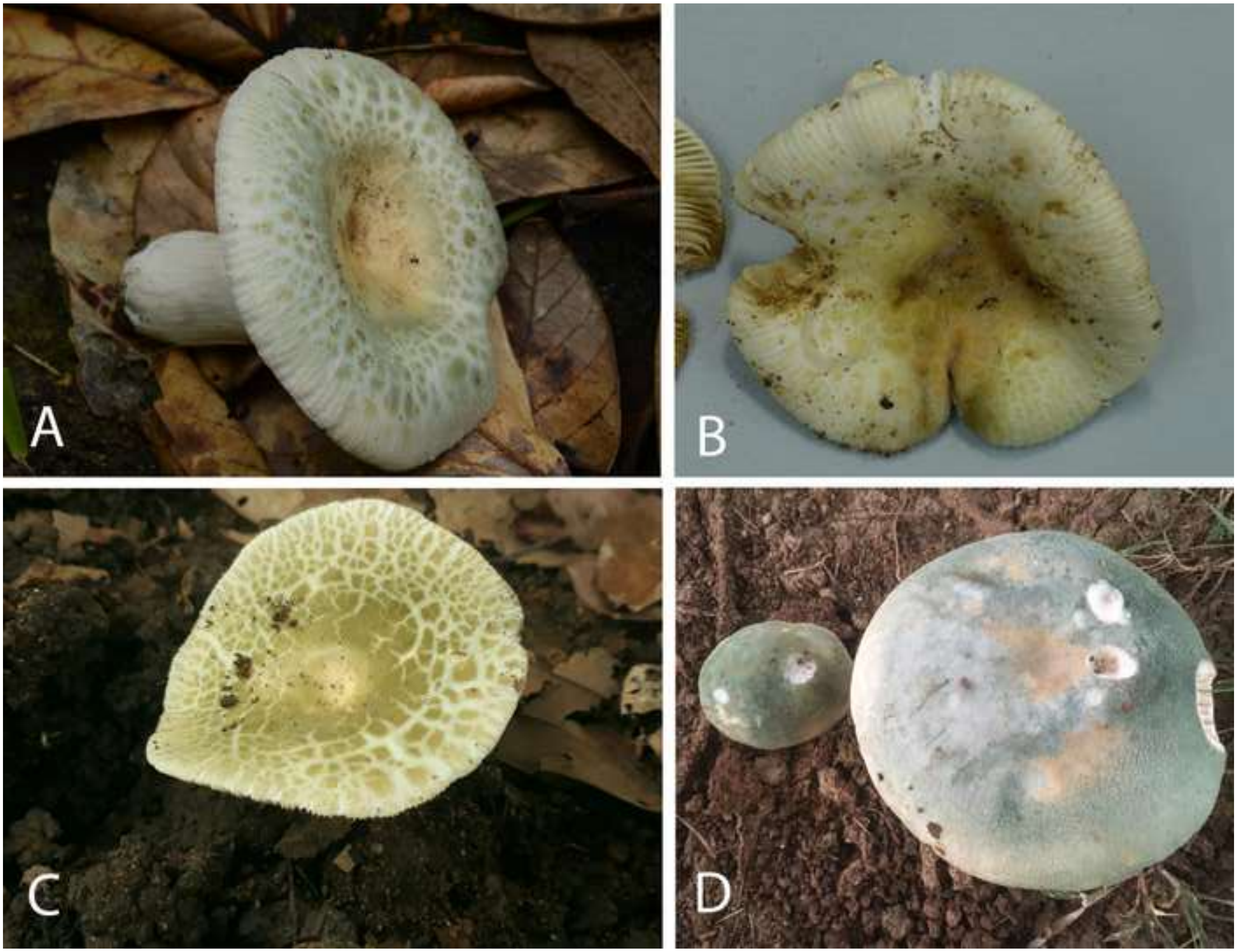

No Comments.

Click here to access/download;Figure;Fig 4. Hymenial characters of R. orientalovirescens.tif

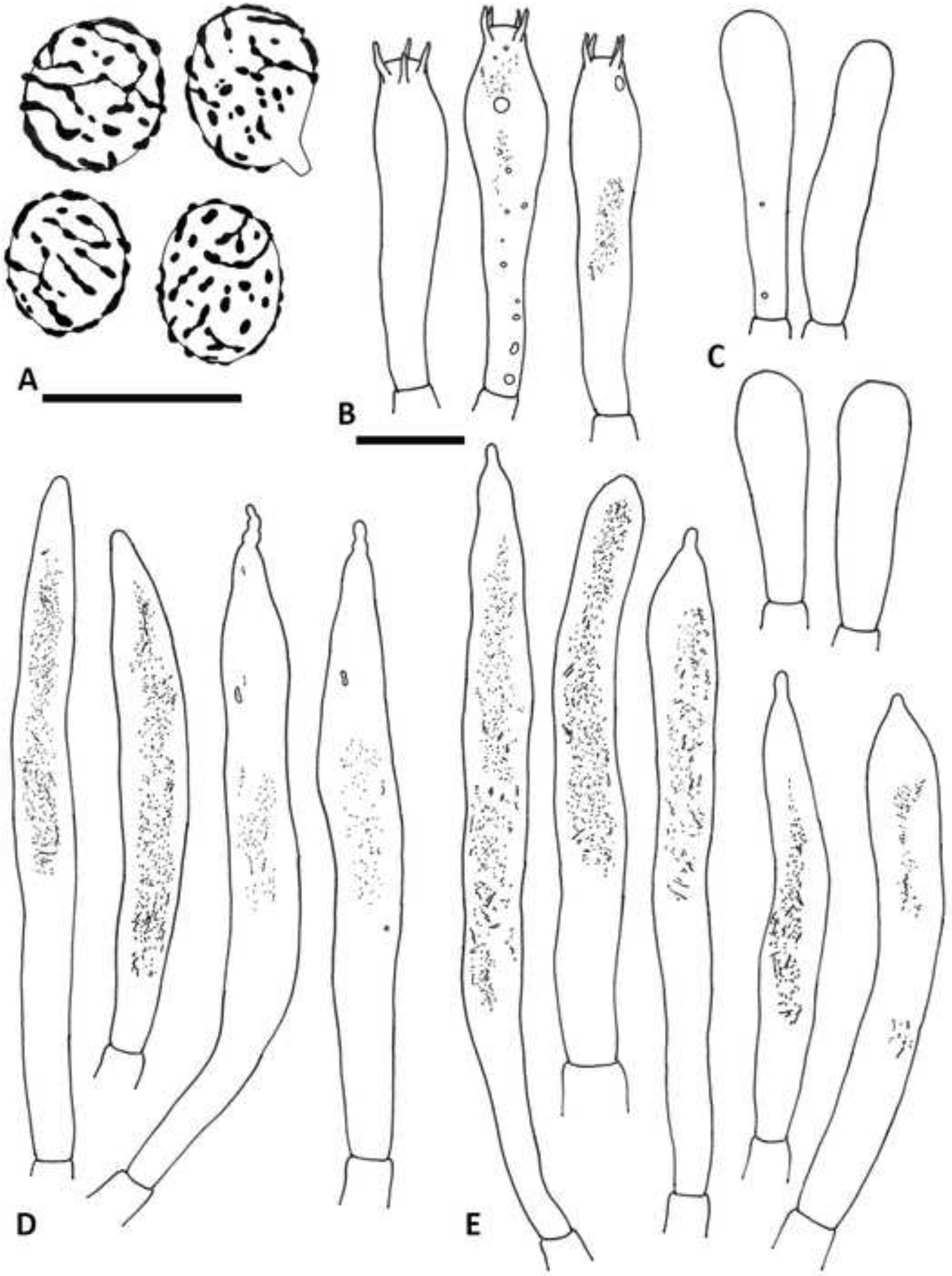

No Comments.

Click here to access/download;Figure;Fig 5. Pileal characters of R. orientalovirescens.tif

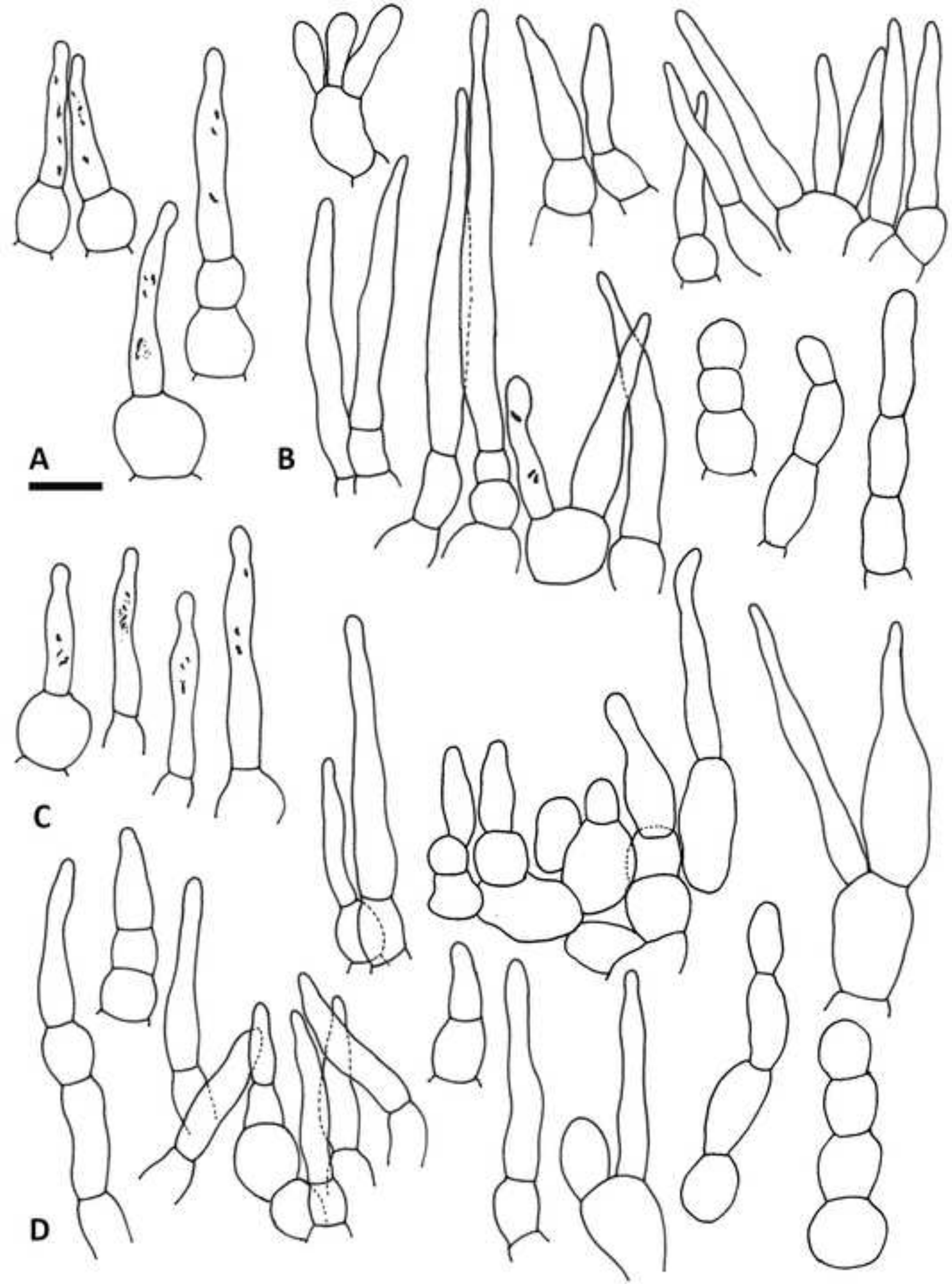

No Comments.

Figure6

Click here to access/download;Figure;Fig. 6 SEM spore R. orientalovirescens.tif

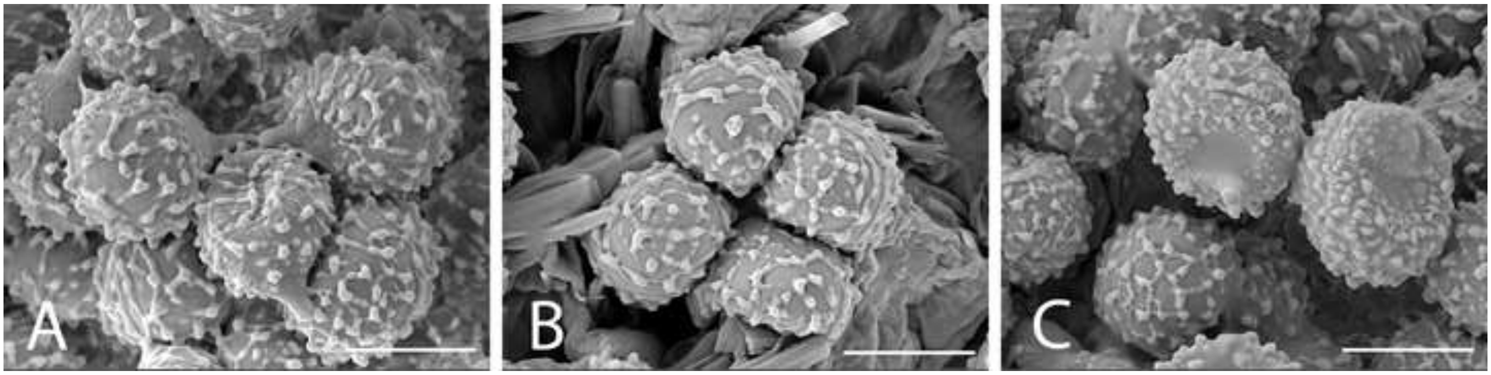

No Comments.

Figure7

Click here to access/download;Figure;Fig. 7 Hymenial elements R. virescens SAVF3978.tif

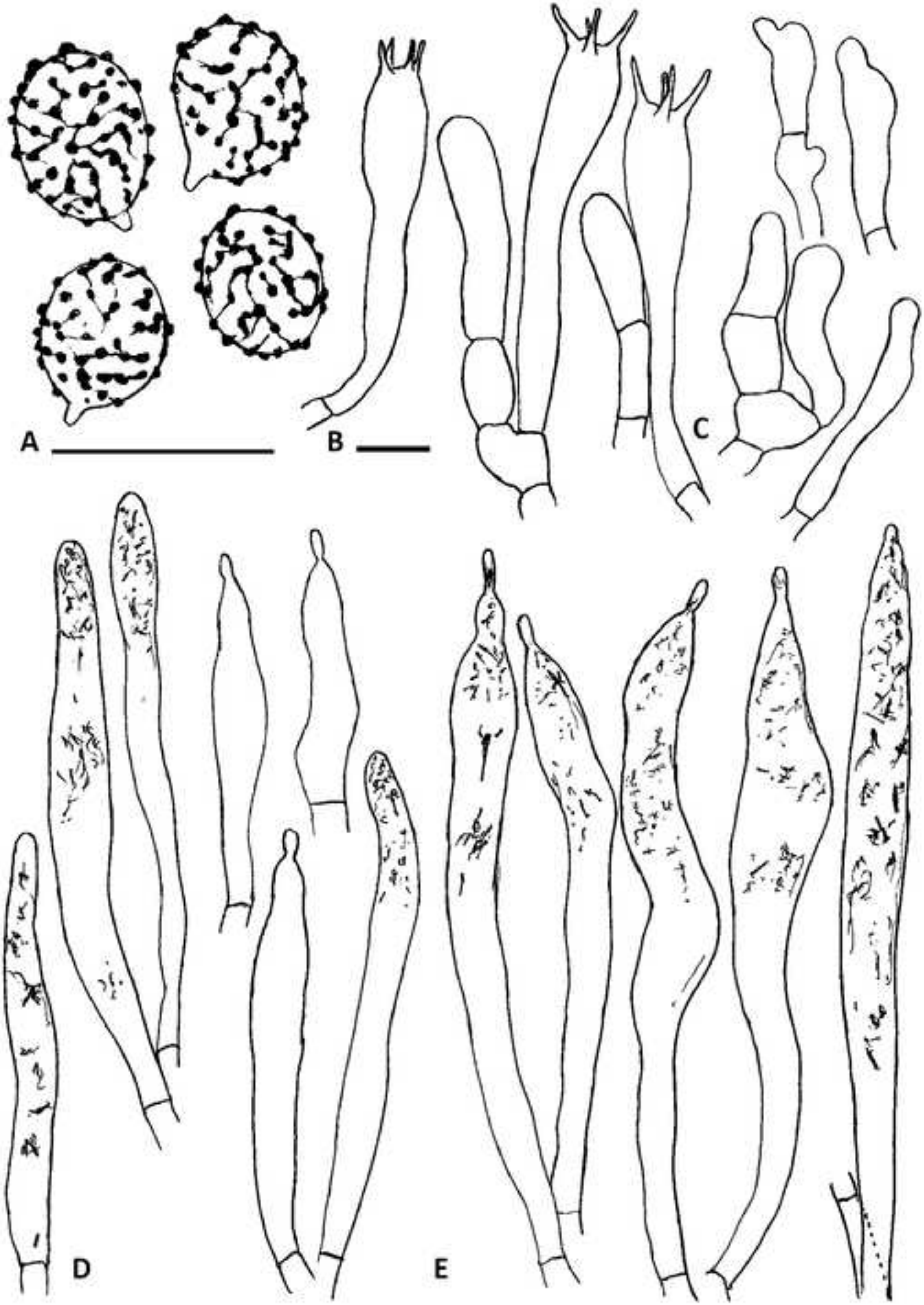

No Comments.

Click here to access/download;Figure;Fig. 8 Pileal elements R. virescens SAVF3978.tif

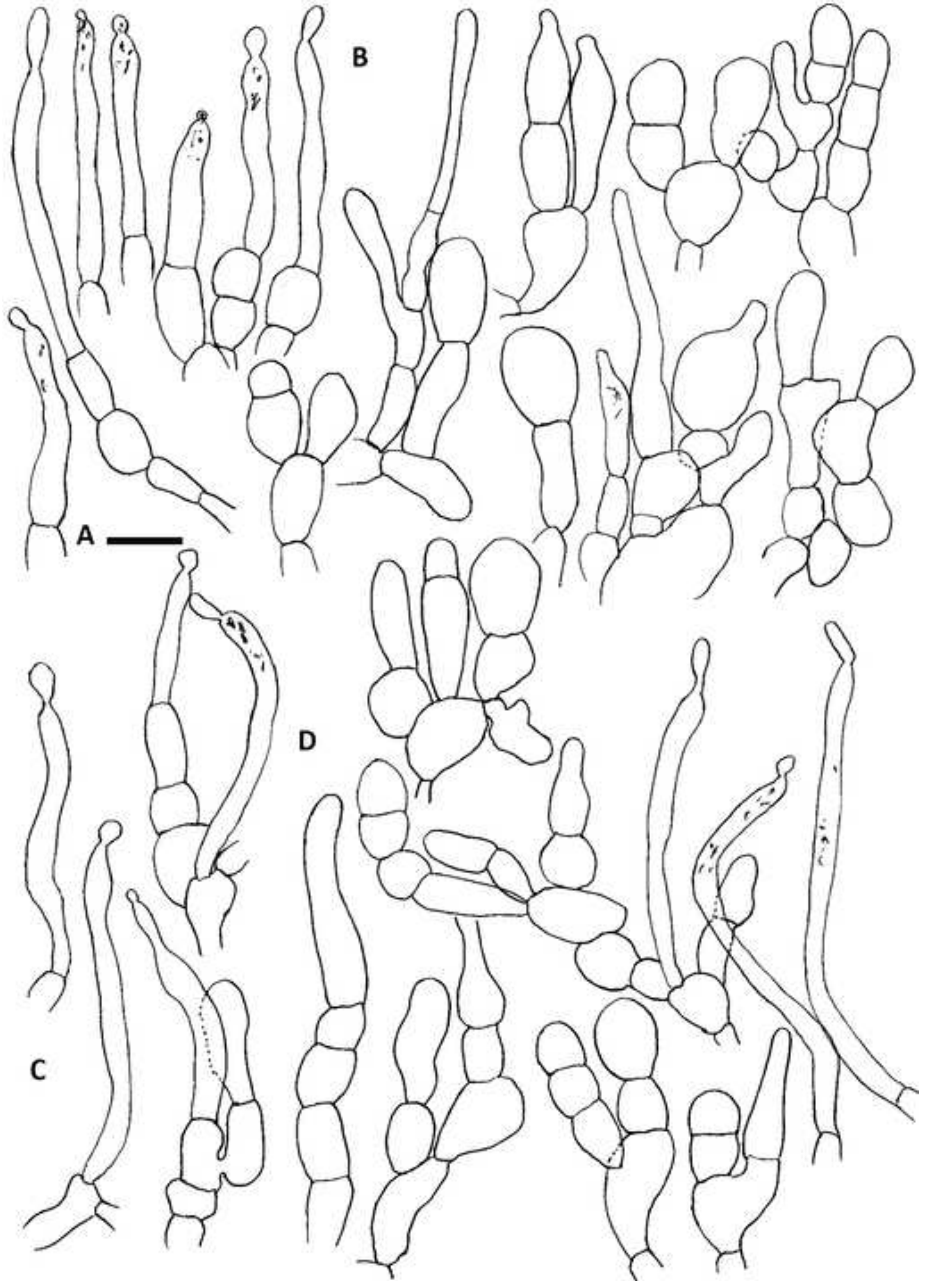

No Comments.
