# Supplementary material for: Russula orientalovirescens sp. nov., a common Southeast Asian edible fungus is different from the European look-alike R. virescens
Source: PLoS One. 2025 Aug 20;20(8):e0322545. doi: 10.1371/journal.pone.0322545 (PMC12367157; doi:10.1371/journal.pone.0322545)
Supplement: Attachment — Submitted filename: Response to Reviewers.docx [file pone.0322545.s004.docx]

**Manuscript PONE-D-24-43558**

**Response to the reviewers’ comments**

**Reviewer #1:** The manuscript dealing with the diversity of green cracking Russulas and the formal description of a new commonly eaten species with major economical importance is a valuable contribution that will in the future help to ensure food safety.
Please pay careful attention to unify formatting styles within the manuscript, especially regarding the two provided species descriptions.

**Reply:** thank you for the positive feedback. We carefully revised the formatting styles.

***Responses to each point raised by reviewer #1***

**Comment 1 (line 62):** change to "Heterophyllidiae"

**Reply:** revised

**Comment 2 (line 153):** add space

**Reply:** added

**Comment 3 (line 224):** Please review the authors abbreviation, which has been unconsistently used in past publications and make a registry with the correct abbreviation in the International Plant Names Index to allow an unambiguous identification of the author.

Wissitrassameewong, K. Wisitrassameewong, Wissitr. or Wisitr.

**Reply:** revised. We used Wisitr., which is the form registered in Index Fungorum and IPNI.

**Comment 4 (line 237):** delete "in"

**Reply:** revised

**Comment 5 (line 240):** please add the colour codes of the chart that is cited in material and methods section similar to the following species description

**Reply:** the colour chart was not used to indicate the colour of the new species. This is inconsistent among both descriptions. We decided to indicate the colours without the colour codes

**Comment 6 (line 244):** taste should be bold, since it is not a subcharacter of odour.

**Reply:** revised

**Comment 7 (line 244):** 1A? 1B?

**Reply:** revised

**Comment 8 (line 245):** "×" is missing

**Reply:** revised

**Comment 9 (line 282):** genera names should be italic

**Reply:** revised

**Comment 10 (line 284):** add "to"

**Reply:** added

**Comment 11 (line 291):** change to "compared"

**Reply:** revised

**Comment 12 (line 315):** bold

**Reply:** revised

**Comment 13 (line 316):** italic

**Reply:** revised

**Comment 14 (line 316):** add taste

**Reply:** added

**Comment 15 (line 331):** it is bold in the previous description.

**Reply:** revised

**Comment 16 (line 332):** later in the text different spelling "thin-walled"

**Reply:** revised

**Comment 17 (line 338):** not bold in previous description.

**Reply:** revised

**Comment 18 (line 351):** The formatting style should be adapted to the format of the previous description.

**Reply:** revised

**Comment 19 (line 355):** genera names in italic

**Reply:** revised

**Comment 20 (line 361):** "Heterophyllidiae"

**Reply:** revised

**Comment 21 (line 373):** change to "exhibits"

**Reply:** revised

**Comment 22 (line 376):** change to "compared"

**Reply:** revised

**Comment 23 (line 395):** change to "indicates"

**Reply:** revised

**Comment 24 (Table 2):** please add information that all values are in µm.

**Reply:** added

**Comment 25 (Figure 4):** change to plural "Basidiospores"

**Reply:** revised

**Comment 26 (Figure 4):** change to plural "Marginal cells"

**Reply:** revised

**Comment 27 (Figure 4):** change to plural "scale bars"

**Reply:** revised

**Comment 28 (Figure 5):** change to plural "hyphal terminations"

**Reply:** revised

**Comment 29 (Figure 7):** see comments Fig. 4

**Reply:** revised

**Comment 30 (Figure 8):** see comments Fig. 5

**Reply:** revised

**Comment 31 (Figure 1 ITS tree):** replace "F. sylvatica" by "Fagus sylvatica" to unify the style.

**Reply:** revised

**Comment 32 (Figure 1 ITS tree):** replace "Slovania" by "Slovenia"

**Reply:** revised

**Comment 33 (Figure 1 ITS tree):** replace "Columbia" by "Colombia"

**Reply:** revised

**Reviewer #2:** The paper by Wisitrassameewong et al. addresses the diversity of commercially valued Russula species within the Virescentinae lineage. Previous studies introduced considerable uncertainties by applying European species names to Asian collections. This study aims to clarify the taxonomy of Thai collections and includes the description of a novel species, Russula orientalovirescens, which is clearly distinct from the European Russula virescens.

At first glance, the study appears to be well-conducted, with adequate sampling and the application of standard phylogenetic methods. Notably, the authors included an analysis of environmental sequences, providing valuable insights into the distribution of the newly described R. orientalovirescens. The results are thoroughly discussed and compared with the current state-of-the-art literature.

However, several issues were raised during the review process:

**Introduction and Hypotheses:** The information provided in the Introduction does not clearly lead to the stated hypotheses. While the hypotheses and aims of the study are scientifically sound, they require clearer and more precise formulation.

**Reply:** We propose this change (Lines 97-104):

Based on the molecular and morphological differences previously reported between the Asian green-areolate collections referred to as *Russula virescens* or *R.* cf. *virescens* in the literature on the one hand, and other green Virescentinae species and the European R. virescens on the other hand, we hypothesized that there is at least one additional Asian species of green-cracking *Russula*. Our aim was to confirm if there is phylogenetic signal (using both ITS and multi-locus data) to distinguish our recent Thai collections from European *R. virescens* and other described similar Asian *Virescentinae* members, and also to identify morphological differences between them in case they represent a distinct species.

**Inconsistencies in Specimen Presentation:** There is significant inconsistency in the documentation of the specimens analyzed for the newly described R. orientalovirescens. The authors stated that four specimens were analyzed, yet:
Only two specimens are listed in Table 1.
Three specimens are depicted in the phylogenetic trees.
Table 2 showcases the morphological variability of three specimens, including specimen OR1607, which is not listed in Table 1 or shown in the phylogenetic trees.
This raises concerns about whether the identity of OR1607 was molecularly confirmed. I strongly encourage the authors to include specimen OR1607 in an additional phylogenetic analysis.

**Reply:** we checked the number of samples in the table 1 and phylogenetic tree carefully. We added the information of OR1607 and OR1619 in the table 1.

**Sequencing Effort:** According to Table 1, only one specimen of R. orientalovirescens (OR1687) had multiple genetic regions sequenced. This contrasts with the more extensive sequencing efforts for European collections of R. mustelina and R. virescens. Similar efforts should be applied to R. orientalovirescens to ensure consistency and robustness of the analyses.

**Reply:** we tried and sequence the ITS, rpb2 and tef1 sequences of OR1607. We could only obtain the ITS sequence.

**Terminology and Descriptions:** Terminology and species descriptions should be more consistent throughout the manuscript (see detailed comments in the attached file).

**Reply:** revised accordingly

**References:** The references require a thorough review for accuracy and completeness.

**Reply:** revised accordingly

Additional minor comments and suggestions are included in the attached file.

***Responses to each point raised by reviewer #2***

**Comment 1 (line 91-92):** Hypothesis does not sound right to me. Since Cao et al. 2013 published their study, multiple species within Virescentinae were described. Additionally, there is Russula viridirubrolimbata which could potentially represent Cao´s "asian virescens". It would be beneficial better elaborate, on which basis you have assumed, that there could be higher diversity.

**Reply:** We rephrased our hypothesis and rationale for it. As explained above in the introduction, in all the Asian green Virescentinae, which do not include R. viridirubrolimbata because the pileus margin of the latter has red colours, the cuticle is cracking at most near the margin. The collections with green and almost entirely areolate cuticle are still referred to as R. virescens or R. cf. virescens despite dissimilarities in ITS sequences.

**Comment 2 (line 92-95):** This aim does not makes a sense to me. Previous phylogenetic studies clearly distinguished asian "virescentinae" from european. You have noted also this fact in the text above, when you state: "Soon it became apparent that at least part of so-called R. virescens in areas distant from Europe represent different species".

Therefore, I do not understand, why is important to distinguish your collections from european Russula virescens rather than closely related Russula viridirubrolimbata

**Reply:** see reply to comment 1

**Comment 3 (line 190):** In the presented tree, there is 42 samples

**Reply:** revised

**Comment 4 (line235):** you should unify units across the manuscript. Description of R. virescens is given in mm instead of cm

**Reply:** revised

**Comment 5 (line 237):** when mature instead of "in when"

**Reply:** revised

**Comment 6 (line 238):** areolae in centre?

**Reply:** revised

**Comment 7 (line 245):** 7.4–8.1–8.8(–9.2) × 6.0–6.4–6.9(–7.2)

**Reply:** revised

**Comment 8 (line 246-250):** about ornamentation are arranged somehow chaotically. I recommend to use same order as for R. virescens

**Reply:** revised

**Comment 9 (line 248):** isolated

**Reply:** revised

**Comment 10 (line 275-276):** information about cystidioid or oleipherous hyphae is absent

**Reply:** added

**Comment 11 (line 418):** superfluous

**Reply:** we prefer to keep it

**Comment 12 (table 2):** suggest to add also comparisons with the other species - R. parvovirescens and R. viridirubrolimbata, from literature

**Reply:** We added data on R. parvovirescens. However, we think that adding R. viridirubrolimbata would not be useful since this species can easily be differentiated from R. virescens based on macromorphology (red colours near pileus margin)

**Comment 13 (table 2):** In the description, you are referring to pleurocystidia as hymenial cystidia on lamellae sided / cheilocystidia on lamellae edges

**Reply:** revised
